# Stable warfarin dose prediction in sub‐Saharan African patients: A machine‐learning approach and external validation of a clinical dose–initiation algorithm

**DOI:** 10.1002/psp4.12740

**Published:** 2021-12-09

**Authors:** Innocent G. Asiimwe, Marc Blockman, Karen Cohen, Clint Cupido, Claire Hutchinson, Barry Jacobson, Mohammed Lamorde, Jennie Morgan, Johannes P. Mouton, Doreen Nakagaayi, Emmy Okello, Elise Schapkaitz, Christine Sekaggya‐Wiltshire, Jerome R. Semakula, Catriona Waitt, Eunice J. Zhang, Andrea L. Jorgensen, Munir Pirmohamed

**Affiliations:** ^1^ Department of Pharmacology and Therapeutics The Wolfson Centre for Personalized Medicine Medical Research Council Centre for Drug Safety Science Institute of Systems, Molecular and Integrative Biology University of Liverpool Liverpool UK; ^2^ Division of Clinical Pharmacology Department of Medicine University of Cape Town Cape Town South Africa; ^3^ Victoria Hospital Internal Medicine Research Initiative Victoria Hospital Wynberg Cape Town South Africa; ^4^ Department of Medicine University of Cape Town Cape Town South Africa; ^5^ Department of Molecular Medicine and Haematology University of the Witwatersrand Johannesburg South Africa; ^6^ Infectious Diseases Institute Makerere University College of Health Sciences Kampala Uganda; ^7^ Metro District Health Services Western Cape Department of Health Cape Town South Africa; ^8^ Uganda Heart Institute Kampala Uganda; ^9^ Department of Molecular Medicine and Hematology Charlotte Maxeke Johannesburg Academic Hospital National Health Laboratory System Complex and University of Witwatersrand Johannesburg South Africa; ^10^ Department of Health Data Science Institute of Population Health Sciences University of Liverpool Liverpool UK

## Abstract

Warfarin remains the most widely prescribed oral anticoagulant in sub‐Saharan Africa. However, because of its narrow therapeutic index, dosing can be challenging. We have therefore (a) evaluated and compared the performance of 21 machine‐learning techniques in predicting stable warfarin dose in sub‐Saharan Black‐African patients and (b) externally validated a previously developed Warfarin Anticoagulation in Patients in Sub‐Saharan Africa (War‐PATH) clinical dose–initiation algorithm. The development cohort included 364 patients recruited from eight outpatient clinics and hospital departments in Uganda and South Africa (June 2018–July 2019). Validation was conducted using an external validation cohort (270 patients recruited from August 2019 to March 2020 in 12 outpatient clinics and hospital departments). Based on the mean absolute error (MAE; mean of absolute differences between the actual and predicted doses), random forest regression (12.07 mg/week; 95% confidence interval [CI], 10.39–13.76) was the best performing machine‐learning technique in the external validation cohort, whereas the worst performing technique was model trees (17.59 mg/week; 95% CI, 15.75–19.43). By comparison, the simple, commonly used regression technique (ordinary least squares) performed similarly to more complex supervised machine‐learning techniques and achieved an MAE of 13.01 mg/week (95% CI, 11.45–14.58). In summary, we have demonstrated that simpler regression techniques perform similarly to more complex supervised machine‐learning techniques. We have also externally validated our previously developed clinical dose–initiation algorithm, which is being prospectively tested for clinical utility.

**Study Highlights**

**WHAT IS THE CURRENT KNOWLEDGE ON THE TOPIC?**

To improve warfarin dose prediction, numerous algorithms, including our previously reported clinical dose–initiation algorithm, have been developed using several techniques.

**WHAT QUESTION DID THIS STUDY ADDRESS?**

Do simpler regression techniques perform similarly to more complex supervised machine‐learning techniques, and what is the external validity of our previously developed clinical dose–initiation model?

**WHAT DOES THIS STUDY ADD TO OUR KNOWLEDGE?**

We have shown that simpler regression techniques perform similarly to more complex supervised machine‐learning techniques and also externally validated our previously developed clinical dose–initiation algorithm.

**HOW MIGHT THIS CHANGE DRUG DISCOVERY, DEVELOPMENT, AND/OR THERAPEUTICS?**

Our short‐term aim is to evaluate the utility of our externally validated clinical dose–initiation algorithm to facilitate dose prediction in sub‐Saharan African patients. In the longer term, simple regression techniques will be used to develop a pharmacogenetic model to further improve the quality of anticoagulation with warfarin.

## INTRODUCTION

Warfarin remains the most commonly prescribed oral anticoagulant in sub‐Saharan Africa.^1^ Dosing is, however, difficult because of its narrow therapeutic index and large intrapatient and interpatient variabilities in dose requirements. Suboptimal dosing can result in thrombotic events (too little warfarin) or hemorrhagic events (too much warfarin). Consequently, warfarin is responsible for the highest number of hospitalizations attributed to preventable adverse drug reactions in South Africa.^2^

To optimize dosing, numerous algorithms have been developed. For example, our recently conducted systematic review identified 433 dosing algorithms, of which 86% were for dose initiation.^3^ In the same review, most (65%) of the algorithms were developed using multiple linear regression techniques, of which ordinary least squares regression was the most common. Several machine‐learning techniques including artificial neural networks, support vector regression, k‐nearest neighbors, regression trees, model trees, least angle regression, least absolute shrinkage and selection operator, multivariate adaptive regression splines, boosted trees, and bagged trees to construct ensemble models, and others were also reported. It is thought that more complex techniques such as artificial neural networks (used to develop 7% of algorithms) and support vector regression (used to develop 6% of algorithms) may outperform multiple linear regression because of the ability to capture very complex relationships.^4^ In our review, multiple linear regression seemed to perform comparably to machine‐learning techniques, although the evidence was inconclusive because of a small number of direct comparisons and infrequent external validations. In addition, none of the other techniques were applied to sub‐Saharan African patients.

The mathematical backgrounds, strengths, and weaknesses of various machine‐learning techniques have been extensively explored^5^, ^6^, ^7^, ^8^, ^9^ and are therefore not included in this report. Rather, the focus of this study was to evaluate and compare the performance (in terms of prediction accuracy, bias, clinical relevance, and risk of underdosing or overdosing) of several machine‐learning techniques with regard to predicting stable warfarin doses in sub‐Saharan black‐African patients. We also externally validated a previously developed Warfarin Anticoagulation in Patients in Sub‐Saharan Africa (War‐PATH) clinical dose–initiation algorithm.^10^

## METHODS

This report follows the Transparent Reporting of a Multivariable Prediction Model for Individual Prognosis or Diagnosis (TRIPOD) statement^11^ (TRIPOD checklist in Table S1).

### Source of data

We used the same development cohort that we previously used to develop the War‐PATH clinical dose–initiation algorithm.^10^ Briefly, this comprised 364 warfarin‐treated patients on stable warfarin dose who were recruited from eight outpatient clinics and hospital departments in Uganda and South Africa between June 2018 and July 2019. Patients (*n* = 270) recruited using the same eligibility criteria between August 2019 and March 2020 formed the external validation cohort (164 were a temporal validation cohort, recruited from the aforementioned eight outpatient clinics/hospital departments,^10^ and 106 were a geographical validation cohort, recruited from the following four additional outpatient clinics/hospital departments: Charlotte Maxeke Johannesburg Academic Hospital, Johannesburg; Michael Mapongwana Community Health Centre, Cape Town; Nolungile Community Health Centre, Cape Town; and, Victoria Hospital, Cape Town). The study complied with all relevant ethical requirements including obtaining institutional review board approvals and individual‐patient informed consent.^10^

### Participants

As previously reported,^10^ consenting adult patients (aged ≥18 years) of self‐reported Black‐African ancestry treated with warfarin for venous thromboembolism, atrial fibrillation, or valvular heart disease were included in the War‐PATH cohort, whereas patients who were unwilling to take part, not on a stable warfarin dose (defined in the “Outcome” sub‐section), or pregnant women and patients with any other contraindications based on clinician judgment were excluded.

### Outcome

The outcome was stable warfarin dose, defined as the same dose prescribed at two consecutive clinic visits in the 12 months preceding recruitment, with the international normalized range (INR) being in therapeutic range at each of those visits.^10^

### Predictors

During the development of the War‐PATH clinical dose–initiation algorithm and based on expert guidance and literature review, we selected seven predictors (country of recruitment, age, sex, weight, target INR range, human immunodeficiency virus [HIV] status, and simvastatin/amiodarone status) for use during the modeling process.^10^ From these, four (age, weight, target INR range, and HIV status) were included in the final War‐PATH clinical dose–initiation algorithm. During the evaluation of the machine‐learning techniques, all of the aforementioned seven predictors were considered.

### Sample size

All available data were used during algorithm development and external validation to maximize the power and generalizability of the results. With eight candidate predictor parameters (HIV status had three factor levels), the study participant‐per‐candidate predictor parameter was 46 (War‐PATH development cohort). The War‐PATH external validation cohort included data from more than 100 individuals, the currently recommended sample size for validation.^12^

### Missing data

We used multivariate imputation by chained equations in R^13^ to impute missing data using all included predictor variables and untransformed stable wafarin dose,^14^ as previously described.^10^ Predictive mean matching was used for continuous variables with logistic regression being used for binary variables. The number of imputed data sets was based on the rule of thumb of having the imputed data sets to at least equal the percentage of incomplete cases.^15^ During model fitting, we were not interested in within‐imputation and between‐imputation variabilities, so we analyzed the imputed data sets as a single/stacked data set^15^ with a length of 1092 (three imputed data sets, only 3% cases were missing data,^10^ multiplied by 364, the number of patients per data set). Once the model was fitted and to ensure the performance standard errors were not underestimated, performance metrics were evaluated in each imputed data set with estimates from the imputed data sets being combined using Rubin's rules.^16^

### Statistical analysis methods

All analyses were conducted in R version 4.0.2^17^ (R code used is available in Text S1, which also includes an example data set and its results).

#### Outcome transformation

To model a proportional/multiplicative scale that is clinically relevant and easy to interpret,^18^, ^19^ nonlinear least squares (log‐log) regression used an untransformed dose, whereas other techniques used a logarithmic transformation.^10^

#### Predictor handling

We neither transformed nor categorized predictor variables. Nonlinearity between continuous predictors and stable dose was previously assessed using restricted cubic splines.^10^ Categorical variables were dummy coded in R.^17^

#### Type of model

We used 21 machine‐learning techniques as detailed in Table S2 (selection was guided by our previous review^3^ and other literature^5^, ^6^, ^7^, ^8^, ^9^).

#### Predictor selection before modeling

As described previously, we used the seven predictors that were previously selected based on expert guidance and literature review.^10^

#### Predictor selection during modeling

Except for the techniques such as least absolute shrinkage and selection operator regression, elastic net, multivariate adaptive regression splines, and tree‐based methods that perform automatic predictor selection during parameter tuning or model fitting, we did not perform predictor selection during modeling.

#### Parameter tuning and uniform shrinkage

For some techniques and to prevent overfitting, the out‐of‐bag bootstrap approach (in which the test performance was computed using the samples not included in each bootstrap iteration)^8^ with 1000 repeats was used to tune model parameters or uniformly shrink model coefficients depending on the technique (Text S1, Table S2). Because the stacked data set that we used comprised three imputed data sets, each patient was represented three times, which meant that bootstrapping this stacked data set would likely result in information from the same patient appearing in both the bootstrap and out‐of‐bag samples. To attain independent bootstrap and out‐of‐bag samples, we therefore performed bootstrapping in each imputed (nonstacked) data set before stacking the resultant bootstrap and out‐of‐bag samples (illustrated under ridge regression in Text S1). During parameter tuning, the performance measure that we used was the mean absolute logarithm of the accuracy ratio, where accuracy ratio is the dose predicted by an algorithm divided by the patient's actual dose.^10^

#### Model validation

Algorithms produced by the 21 machine‐learning techniques, the War‐PATH clinical dose–initiation algorithm^10^ and fixed‐dose initiation (35 mg/week, common practice in sub‐Saharan Africa^20^ in which dosing is started empirically at 5 mg/day) were externally validated using the War‐PATH external validation cohort (*n* = 270).

#### Performance measures

During both model development and validation, we computed prediction accuracy based on the mean absolute error (MAE) and the “unbiased” mean absolute percentage error (“unbiased” MAPE).^10^ These measures are highly correlated, but we present both because unbiased MAPE is the least biased measure, whereas MAE is more widely known/understood. Bias was assessed using a logarithm of the accuracy ratio‐derived measure, whereas clinical relevance was represented by the percentage of patients with ideal dose (defined as predicted dose within 20% of actual dose). Lastly, we also calculated the percentage of patients at risk of underdosing or overdosing (defined as having an actual dose at least 40% lower or higher than the predicted dose, respectively). Justifications for these measures were provided in our previous article.^10^ During external validation of the War‐PATH clinical dose–initiation algorithm, we compared its performance with fixed‐dose initiation, and statistical significance was considered as 95% confidence intervals (CIs) that did not contain zero.

## RESULTS

### Participants

Characteristics of the War‐PATH external validation cohort are presented in Table 1. To enable a quick comparison, the War‐PATH development cohort, which we previously reported,^10^ is also included.

**TABLE 1:** Patient characteristics

Variables	Development cohort, *n* = 364	External validation cohort, *n* = 270
Country of recruitment, *n* (%)		
South Africa	193 (53.0)	171 (63.3)
Uganda	171 (47.0)	99 (36.7)
Age, y		
Median (IQR)	46.0 (34.0–56.7)	45.0 (35.0–59.0)
Sex, *n* (%)		
Female	266 (73.1)	195 (72.2)
Male	98 (26.9)	75 (27.8)
Weight, kg		
Median (IQR)	71.0 (59.4–85.0)	73.4 (60.0–90.0)
Missing, *n* (%)	11 (3.0)	1 (0.4)
INR target range,^a^ *n* (%)		
2.0–3.0	237 (65.1)	181 (67.0)
2.5–3.5	127 (34.9)	89 (33.0)
HIV status, *n* (%)		
Negative	282 (77.5)	207 (76.7)
Positive	59 (16.2)	58 (21.5)
Unknown	23 (6.3)	5 (1.9)
Simvastatin/amiodarone, *n* (%)		
Yes	36 (9.9)	22 (8.1)
No	328 (90.1)	248 (91.9)
Stable warfarin dose, mg/week		
Median (IQR)	35.0 (30.0–52.5)	35.0 (30.0–45.0)

Abbreviations: HIV, human immunodeficiency virus; INR, international normalized range; IQR, interquartile range.

^a^ Those with heart valve disorders have a higher target range (2.5–3.5) than the rest (2.0–3.0) who include those with atrial fibrillation and venous thromboembolism.

### Performances of the machine‐learning techniques

**FIGURE 1:**
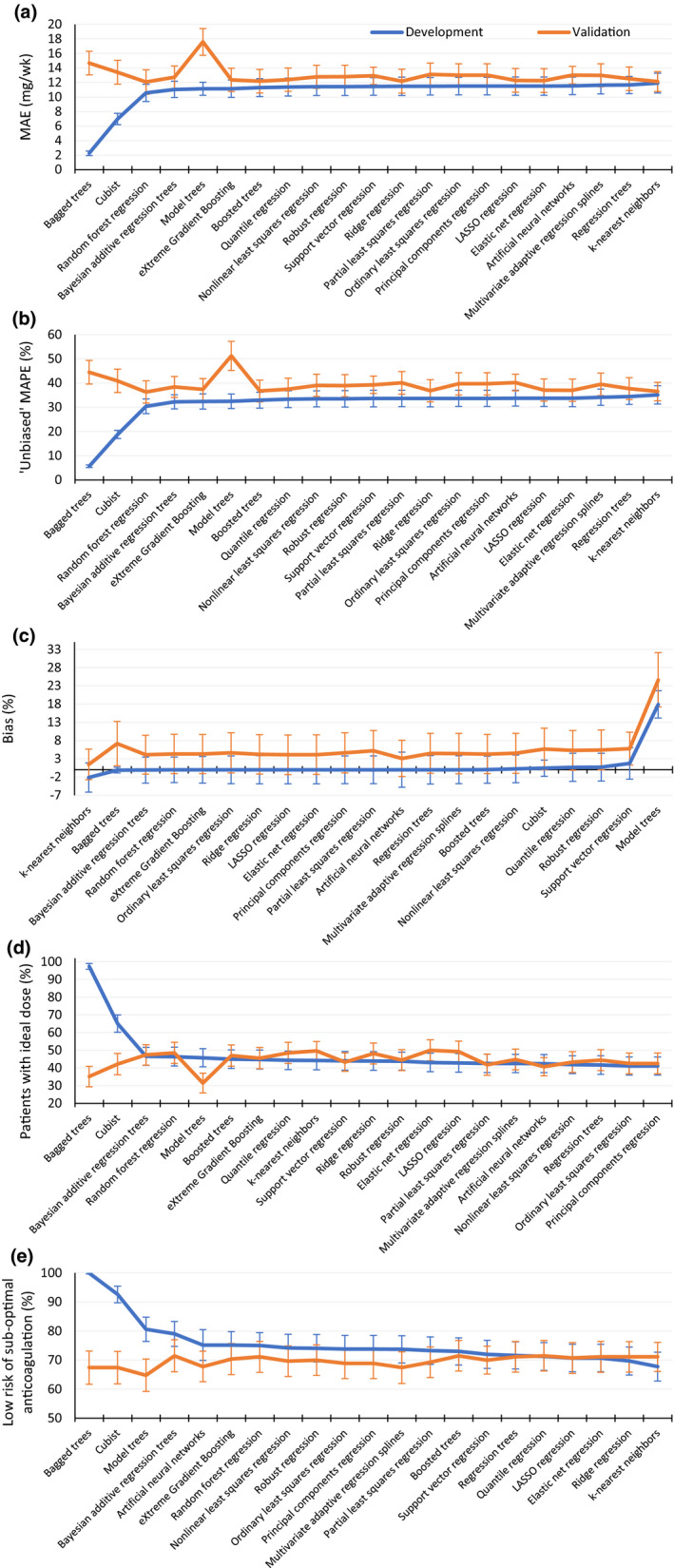
Performance of the various algorithms in the Warfarin Anticoagulation in Patients in Sub‐Saharan Africa development (*N* = 364) and external validation (*N* = 270) cohorts. (a) Mean absolute error (MAE), defined as the mean of absolute differences between the actual and predicted doses. (b) Unbiased mean absolute percentage error (MAPE), where unbiased MAPE = (exp(mean(absolute(log(predicted dose/actual dose))))– 1) × 100. (c) Bias, computed as (exp(mean(log(predicted dose/actual dose))) – 1) × 100, with negative and positive values respectively implying underestimation and overestimation. (d) Percentage of patients with ideal dose, where the ideal dose was defined as a predicted dose within 20% of the actual dose. (e) Low risk of suboptimal anticoagulation, defined as having an actual dose within 40% of the predicted dose. Error bars represent 95% confidence intervals. Based on three imputed data sets (the imputation models incorporated all predictor variables and the stable weekly dose). LASSO, least absolute shrinkage and selection operator

The performances of the various algorithms in the War‐PATH development and external validation cohorts are summarized in Figure 1 and Tables S3 and S4.

#### Predictive accuracy and bias of predictions

In the development data set and in terms of the MAE, bagged trees (2.26 mg/week; 95% CI, 1.95–2.56) and Cubist (6.98 mg/week; 95% CI, 6.20–7.76) were the top performing techniques, with the other techniques having similar performances, which ranged from 10.58 mg/week (95% CI, 9.39–11.76) for random forest regression to 11.91 mg/week (95% CI, 10.55–13.27) for k‐nearest neighbors. In the external validation data set, random forest regression had the lowest MAE (12.07; 95% CI, 10.39–13.76), although this was not very different from most of the other techniques, with the exception of model trees (17.59 mg/week; 95% CI, 15.75–19.43). The simple, commonly used, ordinary least squares regression (13.01 mg/week; 95% CI, 11.45–14.58) was the 15th best performing technique (a joint position with principal components regression), although its performance was similar to that of random forest regression (Figure 1a). A similar trend was observed with the unbiased MAPE (Figure 1b).

Except for model trees, which systematically overpredicted in the development data set (bias of 17.93% above the actual dose; 95% CI, 14.25%–21.73%), all other techniques were unbiased in the development data set. In the external validation set, all techniques overpredicted doses, but only bagged trees, cubist, model trees, support vector regression, quantile regression, and robust regression systematically overpredicted (i.e., had 95% CIs that did not include zero; Figure 1c).

#### Clinical relevance and risk of under‐ or over‐dosing

Similar to predictive accuracy measures, bagged trees (97.34% patients with ideal dose; 95% CI, 95.66%–99.03%) and Cubist (65.02% patients with ideal dose; 95% CI, 60.14%–69.90%) were the top performing models in the development cohort. Other models performed similarly, ranging from 46.61% (95% CI, 41.56%–51.66%) patients with ideal dose for Bayesian additive regression trees to 41.12% (95% CI, 36.01%–46.22%) patients with ideal dose for ordinary least squares/principal components regression. In the external validation cohort, elastic net regression (50.00% patients with ideal dose; 95% CI, 44.11%–55.89%) and k‐nearest neigbors (49.63% patients with ideal dose; 95% CI, 44.35%–54.91%) were the top performers, although this was similar to the performance of other models. Bagged trees and model trees, however, performed poorly (Figure 1d). Regarding the risk of underdosing or overdosing, the best models (those that would put the lowest number of patients at risk of suboptimal anticoagulation) in the validation cohort were Bayesian additive regression trees, boosted trees, and quantile regression, although the differences in performance with other models were slight (Figure 1e).

### External validation of the War‐PATH clinical dose–initiation algorithm

In the external validation cohort (Table S4), the War‐PATH algorithm (12.57 mg/week; 95% CI, 10.99–14.15) had a higher MAE than fixed‐dose initiation (11.91 mg/week; 95% CI, 10.04–13.78), although the difference (0.66 mg/week; 95% CI, −1.79 to 3.11) was not statistically significant. Unbiased MAPE followed a similar trend, with the War‐PATH algorithm predicting doses that were on average within 38.21% (95% CI, 33.80%–42.78%) of the actual doses, which was higher than fixed‐dose initiation by 2.55% (95% CI, −2.30% to 7.41%), a difference that was again not statistically significant. In terms of the extent of the bias of predictions, both the War‐PATH algorithm (bias of 3.84% above the actual dose; 95% CI, −1.40% to 9.36%) and fixed dose (3.34% below the actual dose; 95% CI, −1.97% to 8.38%) were unbiased (95% CIs contained zero).

**FIGURE 2:**
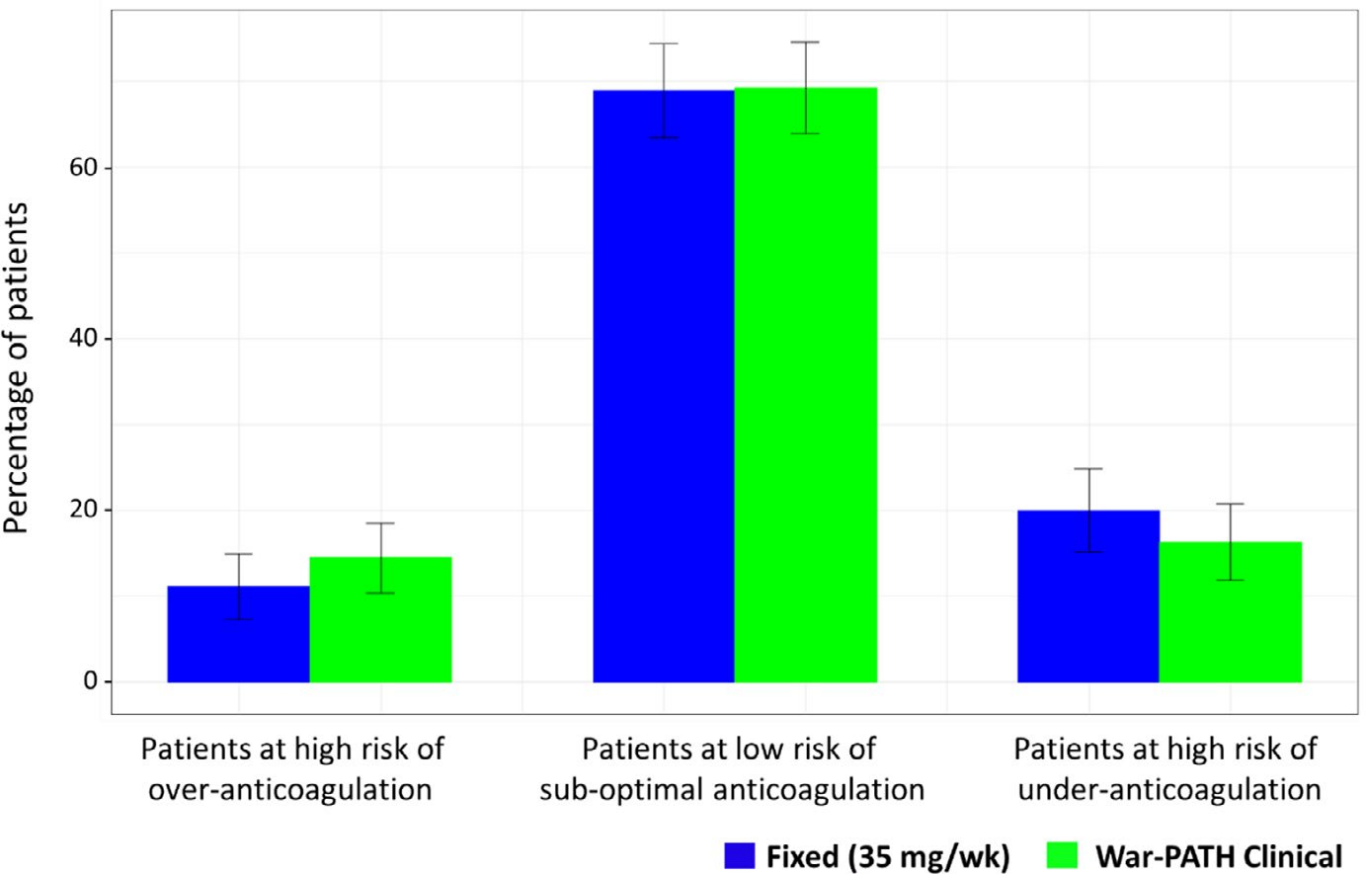
Percentage of patients at risk of suboptimal dosing in the external validation cohort (*N* = 270). Error bars represent 95% confidence intervals. Being at risk of underdosing or overdosing was defined as, respectively, having an actual dose at least 40% lower or higher than the predicted dose. War‐PATH, Warfarin Anticoagulation in Patients in Sub‐Saharan Africa

In terms of clinical relevance, the War‐PATH algorithm (43.33% patients with ideal dose; 95% CI, 37.32%–49.35%) again had a lower performance than fixed‐dose initiation (50.37% patients with ideal dose; 95% CI, 44.52%–56.22%), but the difference (−7.04%; 95% CI, −15.43% to 1.35%) was not statistically significant. With a fixed dose of 35 mg/week, 30/270 and 54/270 patients, respectively, required 40% less warfarin (≤21 mg/week) and 40% more warfarin (≥49 mg/week), which translated into 11.11% and 20.00% of the patients being at high risk of overdosing and underdosing. This implied that 68.89% of the patients were at low risk of suboptimal dosing as shown in Figure 2. Using the same 40% threshold, 14.44% and 16.30% patients would, respectively, be at high risk of overdosing and underdosing with the War‐PATH algorithm, whereas 69.26% would be at low risk of suboptimal dosing. Using the War‐PATH algorithm would therefore increase the percentage of patients at low risk of suboptimal dosing from 68.89% to 69.26% by only 0.37% (95% CI, −7.24% to 7.98%), which is also not statistically significant.

## DISCUSSION

In this study, we compared the performances of 21 machine‐learning techniques with regard to predicting stable warfarin dose in sub‐Saharan Black‐African patients. As previously observed, simple multiple regression techniques such as ordinary least squares regression performed similarly or even better than more complex machine‐learning techniques.^3^ Indeed, the model that consistently performed poorest in the validation cohort was model trees. Importantly, no parameter was tuned for model trees because the M5P function in the Rweka package^21^, ^22^ automatically performs tree pruning during the model‐fitting process. This approach might be suboptimal because, rather than test performance in new individuals (as is done with the out‐of‐bag bootstrap approach), the test performance (MAE in this case) is multiplied by an adjustment factor to cater for optimism.^23^ Mention is made of bagged trees that performed exceedingly well in the development cohort (an MAE of 2.3 mg/week, unbiased MAPE of 5.6%, 97.3% patients with ideal dose, and 99.9% patients at low risk of suboptimal anticoagulation). Although not the best in the validation cohort, the bagged trees’ performance was not far off (e.g., this technique had an MAE of 14.7 mg/week, whereas the best model had an MAE of 12.1 mg/week, and although it would put 33% of patients at risk of suboptimal dosing, the best technique for this metric would still put 29% patients at risk of suboptimal anticoagulation). Bagged trees are a subset of random forest regression (which performed well in both development and validation cohorts)—for bagged trees, the choice of predictor subset is set to the maximum number of predictors, whereas random forest regression can take up any number of predictors, including the maximum.^5^

Our second objective involved externally validating our previous clinical dose–initiation algorithm.^10^ Except for patients at high risk of suboptimal dosing, fixed‐dose initiation performed slightly better than the clinical algorithm, although these differences were not statistically significant. The similar performance between the clinical algorithm and current practice (fixed‐dose initiation) may question the importance of implementing a clinical algorithm. However, algorithm‐based dosing is meant to benefit the minority populations at risk of suboptimal dosing (and who are more likely to suffer from thrombotic/hemorrhagic events) with fixed‐dose initiation, without compromising those not at risk. This implies that the benefits of algorithm‐based dosing can best be realized in populations with a high proportion of at‐risk individuals. Indeed, as we previously reported,^10^ the clinical dose–initiation algorithm significantly decreased the percentage of patients at risk of suboptimal dosing by 8% (95% CI, 2%–14%) and 12% (95% CI, 7%–17%) in the War‐PATH development and the International Warfarin Pharmacogenetics Consortium (IWPC) validation cohorts because the baseline proportions of patients at risk were high (35% and 38%, respectively), with the benefits being more pronounced in the IWPC cohort with more at‐risk individuals. In the external validation cohort, only 31% of patients were at risk of suboptimal anticoagulation with fixed‐dose initiation, which made it harder to detect any benefits associated with clinical dosing. In addition, because unstable patients do not have an outcome variable and are therefore not included during the model development/retrospective validation, the number of at‐risk patients is likely to be underestimated in these studies. With this in mind, the War‐PATH clinical dose–initiation algorithm remains justified for it performs similarly to fixed‐dose initiation even in populations in which fewer patients are in need of algorithm‐based dosing; that is, it does not negatively impact the majority of the population not at risk while it can still benefit those few patients who need it.

In Tables S3 and S4, our previous War‐PATH clinical dose–initiation algorithm and fixed‐dose initiation are presented together with other machine‐learning techniques. Although it was not our aim to compare these two approaches with the machine‐learning techniques, the key differences (specifically between the War‐PATH algorithm and the nonlinear least squares regression technique, which was also used to build the War‐PATH algorithm) are worth mentioning. The differences in performance can be attributed to the aim of the modeling process. Whereas we previously considered model simplicity (fewer predictors) for easier implementation and therefore intentionally performed predictor‐selection during modeling, the current study was not primarily targeted toward implementation, so all candidate predictors (based on clinical expertise and literature review) were considered. This is not to say that the techniques presented here are not implementable. Rather, the majority of the machine‐learning techniques require high computing power and/or online‐based systems, which are not available in many parts of sub‐Saharan Africa. Fortunately, simpler techniques, which can even be translated to paper‐based charts, performed similarly to the more complex techniques. In addition, doses for the War‐PATH algorithm were rounded off to the nearest 2.5 mg tablet to reflect practical use in the respective populations,^10^ which was not done during this machine‐learning exercise. As Tables S3 and S4 show, these differences in approaches nevertheless had minimal impacts on performance.

Our study had several limitations. For example, despite basing on our recent systematic review,^3^ we may have missed some machine‐learning techniques that may be important in the field of warfarin dosing. We also did not consider combined models (ensembles) that include a diverse set of algorithms (e.g., an artificial neural network combined with a decision tree and/or k‐nearest neigbors). Other limitations relate to our eligibility criteria that we previously described^10^ and that include exclusion of unstable patients, nonconsideration of the dose‐revision phase (which made pharmacokinetic‐pharmacodynamic modeling unfeasible in this study), and the exclusion of children. This study also relied on the seven predictor variables that were previously identified, which missed key predictors such as body mass index, adherence, and vitamin K status. In addition, we have not considered genetic predictors in this study, which we will, however, include in future work. The strength of some techniques such as artificial neural networks lie in unmasking complex/unknown relationships between predictor variables and the outcome, so limiting the study variables to those that are established could have limited their advantages. Lastly, our sample size calculations were based on multiple linear regression techniques. Whereas the sample size requirements may be less for some techniques such as least absolute shrinkage and selection operator,^24^ some techniques such as artificial neural networks, support vector regression, and random forest regression may require a participant‐per‐candidate predictor parameter in excess of 200,^25^ which means that for eight candidate predictor parameters, the minimum sample size should have been more than 1600.

In conclusion, the simpler and more widely known multiple linear regression techniques such as ordinary least squares performed similarly to more complex supervised machine‐learning techniques in the War‐PATH cohorts. These simpler techniques were previously used to develop a War‐PATH clinical dose–initiation algorithm, which we have externally validated in this study (and is being prospectively tested for clinical utility). Given that simple techniques can get the job done, we will be using them in achieving our long‐term aim of evaluating the importance of clinical and genetic predictors in improving warfarin dosing in Ugandan and South African patients.

## CONFLICT OF INTEREST

M.P. has received partnership funding for the following: Medical Research Council (MRC) Clinical Pharmacology Training Scheme (cofunded by MRC and Roche, Union Chimique Belge [UCB] Pharma, Eli Lilly, and Novartis); a PhD studentship jointly funded by Engineering and Physical Sciences Research Council and Astra Zeneca; and grant funding from Vistagen Therapeutics. He has also unrestricted educational grant support for the UK Pharmacogenetics and Stratified Medicine Network from Bristol‐Myers Squibb and UCB. He has developed a human leukocyte antigen genotyping panel with MC Diagnostics, but does not benefit financially from this. He is part of the Innovative Medicines Initiative Consortium Accelerating Research and Development for Advanced Therapies (www.ardat.org). C.W. is supported by a Wellcome Clinical Research Career Development Fellowship 222075/Z/20/Z. None of these of funding sources have been used for the current article. All other authors declared no competing interests for this work.

## AUTHOR CONTRIBUTIONS

All authors wrote the manuscript and performed the research. I.G.A., M.B., K.C., C.H., M.L., J.P.M., C.S.‐W., J.R.S., C.W., E.J.Z., A.L.J., and M.P. designed the research. I.G.A. analyzed the data. M.B., K.C., C.C., C.H., B.J., M.L., J.M., J.P.M., D.N., E.O., E.S., C.S.‐W., J.R.S., and C.W. contributed new reagents/analytical tools.

## Supporting information

Supplementary Material
