# Supplementary material for: Stable warfarin dose prediction in sub‐Saharan African patients: A machine‐learning approach and external validation of a clinical dose–initiation algorithm
Source: CPT Pharmacometrics Syst Pharmacol. 2021 Dec 9;11(1):20–9. doi: 10.1002/psp4.12740 (PMC8752108; doi:10.1002/psp4.12740)
Supplement: Supplementary file 1 — Supplementary Material [file PSP4-11-20-s001.docx]

**Warfarin dose prediction in sub-Saharan African patients: a machine learning approach and external validation of a clinical dose-initiation algorithm**

Innocent G. Asiimwe^1^, Marc Blockman^2^, Karen Cohen^2^, Clint Cupido^3^, Claire Hutchinson^1^, Barry Jacobson^4^, Mohammed Lamorde^5^, Jennie Morgan^6^, Johannes P. Mouton^2^, Doreen Nakagaayi^7^, Emmy Okello^7^, Elise Schapkaitz^8^, Christine Sekaggya-Wiltshire^5^, Jerome R. Semakula^5^, Catriona Waitt^1,5^, Eunice J. Zhang^1^, Andrea L. Jorgensen^9,10^, and Munir Pirmohamed^1,10^

^1^The Wolfson Centre for Personalized Medicine, MRC Centre for Drug Safety Science, Department of Pharmacology and Therapeutics, Institute of Systems, Molecular and Integrative Biology, University of Liverpool, United Kingdom. ^2^Division of Clinical Pharmacology, Department of Medicine, University of Cape Town, Cape Town, South Africa. ^3^Victoria Hospital Internal Medicine Research Initiative, Victoria Hospital Wynberg and Department of Medicine, University of Cape Town, Cape Town, South Africa. ^4^Department of Molecular Medicine and Haematology, University of the Witwatersrand, Johannesburg, South Africa. ^5^Infectious Diseases Institute, Makerere University College of Health Sciences, Kampala, Uganda. ^6^Metro District Health Services, Western Cape Department of Health, South Africa. ^7^Uganda Heart Institute, Kampala, Uganda. ^8^Department of Molecular Medicine and Hematology, Charlotte Maxeke Johannesburg Academic Hospital National Health Laboratory System Complex and University of Witwatersrand, Johannesburg, South Africa. ^9^Department of Health Data Science, Institute of Population Health Sciences, University of Liverpool, United Kingdom. ^10^These authors contributed equally.

Correspondence: Innocent G. Asiimwe ([i.asiimwe@liverpool.ac.uk](mailto:i.asiimwe@liverpool.ac.uk), +441517955387); Munir Pirmohamed ([munirp@liverpool.ac.uk](mailto:munirp@liverpool.ac.uk), +44 151 794 5549).

**Table of contents**

**Supplementary Methods………………………………………………………………….……………….………….. 2**

**Supplementary Tables…………………………………………………………………………………….………….. 32**

**Supplementary References..…………………………………………………………..……………………….….. 36**

**Supplementary Methods**

**Text S1: R Code used in analysis**

**##Development dataset**

#Load dataset

WarPATH_data <- read.csv(file.path("C:/Users/oliasiim/Documents/WarPATH_dev.csv"), header = TRUE, stringsAsFactors = T)

#Load mice package (for multiple imputation)

library(mice)

#Impute missing data (3 imputations)

imp <- 3

tempData <- mice(WarPATH_data, m=imp, maxit=50, seed=7)

#Generate bootstrap indices

set.seed(7)

index = matrix(0, nrow = nrow(WarPATH_data), ncol = 1000,

dimnames = list(paste(1: nrow(WarPATH_data)), paste(1:1000)))

for (i in 1:1000){

index[,i] <- sample(1: nrow(WarPATH_data), nrow(WarPATH_data), replace=TRUE)

index

}

#Generate merged dataset

for (k in 1:imp){

data <- complete(tempData, k, include = FALSE)

if(k==1) merged <- data

else merged <- rbind.data.frame(merged,data)

merged

}

#Generate bootstrap indices for merged dataset

set.seed(7)

index_merged = matrix(0, nrow = nrow(merged), ncol = 1000,

dimnames = list(paste(1: nrow(merged)), paste(1:1000)))

for (i in 1:1000){

index_merged[,i] <- sample(1: nrow(merged), nrow(merged), replace=TRUE)

index_merged}

#Model fit and parameter tuning in merged dataset (for ordinary least squares, other techniques shown in a different section)

model_fit <- lm(log(Dose_mg_week) ~ ., merged)

coefs<- model_fit$coefficients

#Shrinkage factor

slope <- c(1:1000)

for (i in 1:1000){

train_boot = merged[index_merged[,i],]

model_boot = lm(log(Dose_mg_week ) ~ ., data = train_boot)

a <- exp(predict(model_boot, merged))

b <- merged$Dose_mg_week

c <- cbind(a,b)

c <- data.frame(c)

pred_model <- lm(b ~ a, c)

summary(pred_model)

slope[i] <- as.numeric(coef(pred_model)["a"])

}

shrinkage_factor <- mean(slope)

shrinkage_factor

#Shrinkage factor 1, so no shrinkage performed

#Performance metrics function

performance_metrics <- function(newdata, predicted_dose){

MAE <- mean(abs(newdata$Dose_mg_week - predicted_dose))

MALAR <- mean(abs(log(predicted_dose/newdata$Dose_mg_week)))

MLR <- mean(log(predicted_dose/newdata$Dose_mg_week))

Ideal_dose <- sum(as.numeric(c(abs(newdata$Dose_mg_week - predicted_dose) < 0.2* newdata$Dose_mg_week)))*100/nrow(newdata)

Under_antign <- sum(as.numeric(c((newdata$Dose_mg_week - predicted_dose) >= 0.4* predicted_dose)))*100/nrow(newdata)

Optimal_antign <- sum(as.numeric(c(abs(newdata$Dose_mg_week - predicted_dose) < 0.4* predicted_dose)))*100/nrow(newdata)

Over_antign <- sum(as.numeric(c(-(newdata$Dose_mg_week - predicted_dose) >= 0.4* predicted_dose)))*100/nrow(newdata)

metrics <- cbind(MAE, MALAR, MLR, Ideal_dose, Under_antign, Optimal_antign, Over_antign)

return(metrics)

}

#Rubin's rules for MAE, Ideal_dose and Under/Optimal/Over-anticoagulation

rubin_1 <- function(results, results_sd, imp){

m <- imp

W <- mean((results_sd)^2)

B <- (1/(m-1))* sum((results - mean(results))^2)

var_performance <- W + (1+(1/m))*B

sd_performance <- sqrt(var_performance)

resultsvar <- mean(results)

CI_performance <- round((resultsvar + c(-1, 1) * qnorm(0.975) * sd_performance), 2)

CI_performance <- paste(CI_performance, collapse = "; ")

resultsvar <- round(resultsvar, 2)

combined_results <- cbind(resultsvar, CI_performance)

return(combined_results)

}

#Rubin's rules for Unbiased MAPE and Bias

rubin_2 <- function(results, results_sd, imp){

m <- imp

W <- mean((results_sd)^2)

B <- (1/(m-1))* sum((results - mean(results))^2)

var_performance <- W + (1+(1/m))*B

sd_performance <- sqrt(var_performance)

resultsvar <- mean(results)

CI_performance <- (resultsvar + c(-1, 1) * qnorm(0.975) * sd_performance)

CI_performance <- round((exp(CI_performance)-1)*100,2)

CI_performance <- paste(CI_performance, collapse = "; ")

resultsvar <- round((exp(resultsvar)-1)*100, 2)

combined_results <- cbind(resultsvar, CI_performance)

return(combined_results)

}

#Prediction function for some techniques

predict.opt1 = function(object, newdata){

measurevar <- colnames(newdata)[ncol(newdata)]

groupvars <- colnames(newdata)[-ncol(newdata)]

form = as.formula(paste(measurevar, paste(groupvars, collapse=" + "), sep=" ~ "))

mat = model.matrix(form, newdata)

coefs = object

xvars = names(coefs)

mat[, xvars] %*% coefs

}

##Results storage vectors

results_vector <- c(1:14)

names(results_vector) <- c("MAE","MAE_CI","Unbiased_MAPE","Unbiased_MAPE_CI","Bias","Bias_CI","Ideal_dose","Ideal_dose_CI","Under","Under_CI","Optimal","Optimal_CI","Over","Over_CI")

for(i in 1:14){

if((i %% 2) == 0) next

a <- c(1:imp)

assign(paste("results",names(results_vector)[i],sep="_"),get("a"))

assign(paste("results_sd",names(results_vector)[i],sep="_"),get("a"))

}

#Bootstrap storage vectors

for(i in 1:14){

if((i %% 2) == 0) next

a <- c(1:1000)

assign(paste("bootstrap",names(results_vector)[i],sep="_"),get("a"))

}

#Performance

for (j in 1:imp){

data_dev <- complete(tempData, j, include = FALSE)

predicted_dose <- exp(predict(model_fit, data_dev))

original_metrics <- performance_metrics(data_dev, predicted_dose)

for (i in 1:1000){

data_boot = data_dev[index[,i],]

predicted_dose <- exp(predict(model_fit, data_boot))

bootstrap_metrics <- performance_metrics(data_boot, predicted_dose)

bootstrap_MAE[i] <- bootstrap_metrics[1]

bootstrap_Unbiased_MAPE[i] <- bootstrap_metrics[2]

bootstrap_Bias[i] <- bootstrap_metrics[3]

bootstrap_Ideal_dose[i] <- bootstrap_metrics[4]

bootstrap_Under[i] <- bootstrap_metrics[5]

bootstrap_Optimal[i] <- bootstrap_metrics[6]

bootstrap_Over[i] <- bootstrap_metrics[7]

bootstrap_MAE

bootstrap_Unbiased_MAPE

bootstrap_Bias

bootstrap_Ideal_dose

bootstrap_Under

bootstrap_Optimal

bootstrap_Over

}

results_MAE[j] <- original_metrics[1]

results_sd_MAE[j] <- sd(bootstrap_MAE)

results_Unbiased_MAPE[j] <- original_metrics[2]

results_sd_Unbiased_MAPE[j] <- sd(bootstrap_Unbiased_MAPE)

results_Bias[j] <- original_metrics[3]

results_sd_Bias[j] <- sd(bootstrap_Bias)

results_Ideal_dose[j] <- original_metrics[4]

results_sd_Ideal_dose[j] <- sd(bootstrap_Ideal_dose)

results_Under[j] <- original_metrics[5]

results_sd_Under[j] <- sd(bootstrap_Under)

results_Optimal[j] <- original_metrics[6]

results_sd_Optimal[j] <- sd(bootstrap_Optimal)

results_Over[j] <- original_metrics[7]

results_sd_Over[j] <- sd(bootstrap_Over)

}

results_vector[1] <- rubin_1(results_MAE,results_sd_MAE,imp)[1]

results_vector[2] <- rubin_1(results_MAE,results_sd_MAE,imp)[2]

results_vector[3] <- rubin_2(results_Unbiased_MAPE,results_sd_Unbiased_MAPE,imp)[1]

results_vector[4] <- rubin_2(results_Unbiased_MAPE,results_sd_Unbiased_MAPE,imp)[2]

results_vector[5] <- rubin_2(results_Bias,results_sd_Bias,imp)[1]

results_vector[6] <- rubin_2(results_Bias,results_sd_Bias,imp)[2]

results_vector[7] <- rubin_1(results_Ideal_dose,results_sd_Ideal_dose,imp)[1]

results_vector[8] <- rubin_1(results_Ideal_dose,results_sd_Ideal_dose,imp)[2]

results_vector[9] <- rubin_1(results_Under,results_sd_Under,imp)[1]

results_vector[10] <- rubin_1(results_Under,results_sd_Under,imp)[2]

results_vector[11] <- rubin_1(results_Optimal,results_sd_Optimal,imp)[1]

results_vector[12] <- rubin_1(results_Optimal,results_sd_Optimal,imp)[2]

results_vector[13] <- rubin_1(results_Over,results_sd_Over,imp)[1]

results_vector[14] <- rubin_1(results_Over,results_sd_Over,imp)[2]

#write.csv(results_vector, file = "Model_dev.csv")

**##Validation dataset**

#Load dataset

data_val <- read.csv(file.path("C:/Users/oliasiim/Documents/WarPATH_val.csv"), header = TRUE, stringsAsFactors = T)

#Impute missing data (3 imputations)

imp <- 3

tempData1 <- mice(data_val, m=imp, maxit=50, seed=7)

#Generate bootstrap indices

set.seed(7)

index2 <- matrix(0, nrow = nrow(data_val), ncol = 1000,

dimnames = list(paste(1: nrow(data_val)), paste(1:1000)))

for (i in 1:1000){

index2[,i] <- sample(1: nrow(data_val), nrow(data_val), replace=TRUE)

index2

}

#Performance

for (j in 1:imp){

data_val <- complete(tempData1, j, include = FALSE)

predicted_dose <- exp(predict(model_fit, data_val))

original_metrics <- performance_metrics(data_val, predicted_dose)

for (i in 1:1000){

data_boot = data_val[index2[,i],]

predicted_dose <- exp(predict(model_fit, data_boot))

bootstrap_metrics <- performance_metrics(data_boot, predicted_dose)

bootstrap_MAE[i] <- bootstrap_metrics[1]

bootstrap_Unbiased_MAPE[i] <- bootstrap_metrics[2]

bootstrap_Bias[i] <- bootstrap_metrics[3]

bootstrap_Ideal_dose[i] <- bootstrap_metrics[4]

bootstrap_Under[i] <- bootstrap_metrics[5]

bootstrap_Optimal[i] <- bootstrap_metrics[6]

bootstrap_Over[i] <- bootstrap_metrics[7]

bootstrap_MAE

bootstrap_Unbiased_MAPE

bootstrap_Bias

bootstrap_Ideal_dose

bootstrap_Under

bootstrap_Optimal

bootstrap_Over

}

results_MAE[j] <- original_metrics[1]

results_sd_MAE[j] <- sd(bootstrap_MAE)

results_Unbiased_MAPE[j] <- original_metrics[2]

results_sd_Unbiased_MAPE[j] <- sd(bootstrap_Unbiased_MAPE)

results_Bias[j] <- original_metrics[3]

results_sd_Bias[j] <- sd(bootstrap_Bias)

results_Ideal_dose[j] <- original_metrics[4]

results_sd_Ideal_dose[j] <- sd(bootstrap_Ideal_dose)

results_Under[j] <- original_metrics[5]

results_sd_Under[j] <- sd(bootstrap_Under)

results_Optimal[j] <- original_metrics[6]

results_sd_Optimal[j] <- sd(bootstrap_Optimal)

results_Over[j] <- original_metrics[7]

results_sd_Over[j] <- sd(bootstrap_Over)

}

results_vector[1] <- rubin_1(results_MAE,results_sd_MAE,imp)[1]

results_vector[2] <- rubin_1(results_MAE,results_sd_MAE,imp)[2]

results_vector[3] <- rubin_2(results_Unbiased_MAPE,results_sd_Unbiased_MAPE,imp)[1]

results_vector[4] <- rubin_2(results_Unbiased_MAPE,results_sd_Unbiased_MAPE,imp)[2]

results_vector[5] <- rubin_2(results_Bias,results_sd_Bias,imp)[1]

results_vector[6] <- rubin_2(results_Bias,results_sd_Bias,imp)[2]

results_vector[7] <- rubin_1(results_Ideal_dose,results_sd_Ideal_dose,imp)[1]

results_vector[8] <- rubin_1(results_Ideal_dose,results_sd_Ideal_dose,imp)[2]

results_vector[9] <- rubin_1(results_Under,results_sd_Under,imp)[1]

results_vector[10] <- rubin_1(results_Under,results_sd_Under,imp)[2]

results_vector[11] <- rubin_1(results_Optimal,results_sd_Optimal,imp)[1]

results_vector[12] <- rubin_1(results_Optimal,results_sd_Optimal,imp)[2]

results_vector[13] <- rubin_1(results_Over,results_sd_Over,imp)[1]

results_vector[14] <- rubin_1(results_Over,results_sd_Over,imp)[2]

#write.csv(results_vector, file = "Model_val.csv")

**##Model fit and parameter tuning for other techniques in the merged dataset**

#Nonlinear least squares regression

#Generate index_merged2 for 2000 columns

set.seed(7)

index_merged2 = matrix(0, nrow = nrow(merged), ncol = 2000,

dimnames = list(paste(1: nrow(merged)), paste(1:2000)))

for (i in 1:2000){

index_merged2[,i] <- sample(1: nrow(merged), nrow(merged), replace=TRUE)

index_merged2}

#Generating model

X1 <- merged[, -ncol(merged), drop = FALSE]

X1 <- model.matrix(~ ., data = X1)

measurevar <- "log(Dose_mg_week) ~ log("

groupvars <- colnames(X1)

if("GenderMale" %in% groupvars) replace(groupvars, groupvars == "GenderMale", "as.numeric(GenderMale)")

if("Target_INRThree" %in% groupvars) replace(groupvars, groupvars == "Target_INRThree", "as.numeric(Target_INRThree)")

if("HIV_statusPositive" %in% groupvars) replace(groupvars, groupvars == "HIV_statusPositive", "as.numeric(HIV_statusPositive)")

if("HIV_statusUnknown" %in% groupvars) replace(groupvars, groupvars == "HIV_statusUnknown", "as.numeric(HIV_statusUnknown)")

if("Simvastatin_amiodaroneYes" %in% groupvars) replace(groupvars, groupvars == "Simvastatin_amiodaroneYes", "as.numeric(Simvastatin_amiodaroneYes)")

lets <- c("a", "b", "c", "d", "e", "f", "g", "h", "i", "j", "k", "l")

lets <- lets[1:length(groupvars)]

groupvars <- paste(lets, groupvars, sep=" * ")

groupvars[1] <- lets[1]

form = as.formula(paste(measurevar, paste(groupvars, collapse=" + "),")"))

theta <- lm(Dose_mg_week ~., merged)$coefficients

names(theta) <- lets

data1 <- model.matrix(~.,merged)

data1 <- as.data.frame(data1)

data1 <- data1[,-1]

opt1_par <- as.vector(summary(nls(form, start = theta, data1))$coefficients[,1])

names(opt1_par) <- colnames(X1)

opt1_par

new_coef_rubin <- opt1_par

#Computing shrinkage factor

slope <- c(1:2000)

predict_check1 <- c(1:2000)

predict_check2 <- c(1:2000)

for (i in 1:2000){

train_boot = merged[index_merged2[,i],] #use an index merged comprising 2000 instead of 1000 columns as some errors may arise when the algorithm doesn’t converge

X1 <- train_boot[, -ncol(train_boot), drop = FALSE]

X1 <- model.matrix(~ ., data = X1)

measurevar <- "log((Dose_mg_week)) ~ log("

groupvars <- colnames(X1)

if("GenderMale" %in% groupvars) replace(groupvars, groupvars == "GenderMale", "as.numeric(GenderMale)")

if("Target_INRThree" %in% groupvars) replace(groupvars, groupvars == "Target_INRThree", "as.numeric(Target_INRThree)")

if("HIV_statusPositive" %in% groupvars) replace(groupvars, groupvars == "HIV_statusPositive", "as.numeric(HIV_statusPositive)")

if("HIV_statusUnknown" %in% groupvars) replace(groupvars, groupvars == "HIV_statusUnknown", "as.numeric(HIV_statusUnknown)")

if("Simvastatin_amiodaroneYes" %in% groupvars) replace(groupvars, groupvars == "Simvastatin_amiodaroneYes", "as.numeric(Simvastatin_amiodaroneYes)")

lets <- c("a", "b", "c", "d", "e", "f", "g", "h", "i", "j", "k", "l")

lets <- lets[1:length(groupvars)]

groupvars <- paste(lets, groupvars, sep=" * ")

groupvars[1] <- lets[1]

form = as.formula(paste(measurevar, paste(groupvars, collapse=" + "),")"))

theta <- lm((Dose_mg_week) ~., train_boot)$coefficients

names(theta) <- lets

train_boot1 <- model.matrix(~.,train_boot)

train_boot1 <- as.data.frame(train_boot1)

train_boot1 <- train_boot1[,-1]

tryCatch({

predict_check1[i] <- 2000 * as.vector(summary(nls(form, start = theta, train_boot1))$coefficients[1,1])

}, error=function(e){})

predict_check1

if (predict_check1[i] > 2000){

opt1_par <- as.vector(summary(nls(form, start = theta, train_boot1))$coefficients[,1])

names(opt1_par) <- colnames(X1)

a1 <- predict.opt1(opt1_par, merged)

a1 <- as.vector(a1)

b1 <- merged$Dose_mg_week

c <- cbind(a1,b1)

c <- data.frame(c)

X1 <- c[,1]

#Add ones to X1 for the intercept

X1 <- cbind(1,X1)

colnames(X1) <- c("(Intercept)", "a1")

measurevar <- "log((b1)) ~ log("

groupvars <- colnames(X1)

lets <- c("a", "b", "c", "d", "e", "f", "g", "h", "i")

lets <- lets[1:length(groupvars)]

groupvars <- paste(lets, groupvars, sep=" * ")

groupvars[1] <- lets[1]

form = as.formula(paste(measurevar, paste(groupvars, collapse=" + "),")"))

theta <- lm(b1 ~ a1, c)$coefficients

names(theta) <- lets

c <- model.matrix(~.,c)

c <- as.data.frame(c)

c <- c[,-1]

tryCatch({

predict_check2[i] <- 2000 * as.vector(summary(nls(form, start = theta, c))$coefficients[1,1])

}, error=function(e){})

predict_check2

if (predict_check2[i] > 2000){

opt1_par <- as.vector(summary(nls(form, start = theta, c))$coefficients[,1])

slope[i] <- as.numeric(opt1_par[2])

slope

}

else {

slope[i] <- NA

slope

}

}

else {

slope[i] <- NA

slope

}

}

shrinkage_factor <- mean(slope, na.rm = T)

#Uniform shrinkage (multiply all coefficients by this shrinkage factor, except intercept).

new_coef_shrunk <- new_coef_rubin

new_coef_shrunk [-1] <- new_coef_rubin[-1] * shrinkage_factor

new_coef_shrunk [1] <- 0

#Re-estimating the intercept

predict.rubin = function(object, newdata){

form = as.formula((Dose_mg_week) ~ Country_recruitment + Age_years + Weight_kg + Gender + Target_INR + HIV_status + Simvastatin_amiodarone)

mat = model.matrix(form, newdata)

coefs = object

xvars = names(coefs)

mat[, xvars] %*% coefs

}

y <- predict.rubin(new_coef_rubin, merged)

mx <- predict.rubin(new_coef_shrunk, merged)

new_intercept <- mean(y - mx)

new_coef_shrunk[1] <- new_intercept

#Final model

opt1_par <- new_coef_shrunk

#During prediction in the development dataset, predicted dose is obtained by predicted_dose <- predict.opt1(opt1_par, data_dev). A similar formula is used for the bootstrap/validation datasets.

#Quantile regression

#Loading package and generating model

library(quantreg)

model_fit <- rq(log(Dose_mg_week) ~ ., tau = 0.5, merged)

coefs<- model_fit$coefficients

#Shrinkage factor

slope <- c(1:1000)

for (i in 1:1000){

train_boot = merged[index_merged[,i],]

model_boot = rq(log(Dose_mg_week ) ~ ., tau = 0.5, data = train_boot)

a <- exp(predict(model_boot, merged))

b <- merged$Dose_mg_week

c <- cbind(a,b)

c <- data.frame(c)

pred_model <- rq(b ~ a, tau = 0.5, c)

summary(pred_model)

slope[i] <- as.numeric(coef(pred_model)["a"])

}

shrinkage_factor <- mean(slope)

#Uniform shrinkage (multiply all coefficients by this shrinkage factor, except intercept).

new_coefs <- coefs

new_coefs[-1] <- coefs[-1] * shrinkage_factor

new_coefs[1] <- 0

#Re-estimating the intercept

predict.rubin = function(object, newdata){

form = as.formula((Dose_mg_week) ~ Country_recruitment + Age_years + Weight_kg + Gender + Target_INR + HIV_status + Simvastatin_amiodarone)

mat = model.matrix(form, newdata)

coef = object

xvars = names(coef)

mat[, xvars] %*% coef

}

y <- predict.rubin(coefs, merged)

mx <- predict.rubin(new_coefs, merged)

new_intercept <- mean(y - mx)

new_coefs[1] <- new_intercept

#Final model

opt1_par <- new_coefs

#During prediction in the development dataset, predicted dose is obtained by predicted_dose <- exp(predict.opt1(opt1_par, data_dev)). A similar formula is used for the bootstrap/validation datasets.

#Robust regression

#Loading package and generating model

library(MASS)

model_fit <- rlm(log(Dose_mg_week) ~ ., merged)

coefs<- model_fit$coefficients

#Shrinkage factor

slope <- c(1:1000)

for (i in 1:1000){

train_boot = merged[index_merged[,i],]

model_boot = rlm(log(Dose_mg_week ) ~ ., tau = 0.5, data = train_boot)

a <- exp(predict(model_boot, merged))

b <- merged$Dose_mg_week

c <- cbind(a,b)

c <- data.frame(c)

pred_model <- rlm(b ~ a, c)

summary(pred_model)

slope[i] <- as.numeric(coef(pred_model)["a"])

}

shrinkage_factor <- mean(slope)

#Uniform shrinkage (multiply all coefficients by this shrinkage factor, except intercept).

new_coefs <- coefs

new_coefs[-1] <- coefs[-1] * shrinkage_factor

new_coefs[1] <- 0

#Re-estimating the intercept

#Using our coefficients

predict.rubin = function(object, newdata){

form = as.formula((Dose_mg_week) ~ Country_recruitment + Age_years + Weight_kg + Gender + Target_INR + HIV_status + Simvastatin_amiodarone)

mat = model.matrix(form, newdata)

coef = object

xvars = names(coef)

mat[, xvars] %*% coef

}

y <- predict.rubin(coefs, merged)

mx <- predict.rubin(new_coefs, merged)

new_intercept <- mean(y - mx)

new_coefs[1] <- new_intercept

new_coefs

#Final model

opt1_par <- new_coefs

#During prediction in the development dataset, predicted dose is obtained by predicted_dose <- exp(predict.opt1(opt1_par, data_dev)). A similar formula is used for the bootstrap/validation datasets.

#Ridge regression

#Load package

library(glmnet)

#Tuning lambda

X = model.matrix(Dose_mg_week ~ ., merged)[, -1]

y = log(merged$Dose_mg_week)

model_Ridge=glmnet(X,y,alpha=0)

lambda_seq <- model_Ridge$lambda

results <- c(1:length(lambda_seq))

for (i in 1:length(lambda_seq)){

OOB = c(1:1000) #OOB is out-of-bag bootstrap performance

for (j in 1:1000){

for (m in 1:imp){

data <- complete(tempData, m, include = FALSE) #obtaining the m^th^ imputed non-stacked dataset

dataj <- data[index[,j],] #obtaining a bootstrap of specific patients (those who appear in index j)

if(m==1) train_boot <- dataj

else train_boot <- rbind.data.frame(train_boot,dataj) #merging the bootstrap datasets

train_boot #the stacked bootstrapped datasets

}

for (m in 1:imp){

data <- complete(tempData, m, include = FALSE) #obtaining the m^th^ imputed non-stacked dataset

dataj <- data[-index[,j],] #obtaining the out-of-bag samples (patients who do not appear in index j)

if(m==1) validate_boot <- dataj

else validate_boot <- rbind.data.frame(validate_boot,dataj) #merging the out-of-bag samples

validate_boot #the stacked out-of-bag samples

}

X1 = model.matrix(Dose_mg_week ~ ., train_boot)[, -1]

X2 = model.matrix(Dose_mg_week ~ ., validate_boot)[, -1]

model_fit=glmnet(X1, log(train_boot$Dose_mg_week),alpha=0)

predicted_dose <- exp(predict(model_fit, newx = X2, s= lambda_seq[i]))

test_performance <- mean(abs(log(predicted_dose/validate_boot$Dose_mg_week)))

OOB[j] = test_performance

OOB

}

results[i] <- mean(OOB)

results

}

results

plot(lambda_seq, results)

#which.min(results) == 66

#Model fit in merged dataset

lambda_best <- lambda_seq[which.min(results)]

X = model.matrix(Dose_mg_week ~ ., merged)[, -1]

y = log(merged$Dose_mg_week)

model_Ridge=glmnet(X,y,alpha=0)

#During prediction in the development dataset, predicted dose is obtained by predicted_dose <- exp(predict (model_Ridge, newx = X, s = lambda_best)), where X <- model.matrix(Dose_mg_week ~ ., data_dev)[, -1]. Similar formulae are used for the bootstrap/validation datasets.

#LASSO regression

#Same as for ridge regression, except that alpha = 1

# Elastic net regression

#Same as for ridge regression, except that alpha = 0.5

#Principal components regression

#Load package

library (pls)

#Tuning number of principal components

set.seed(7)

model_PCR <- pcr(log(Dose_mg_week) ~ ., data = merged, scale = TRUE)

results <- c(1:model_PCR$ncomp)

for (i in 1:model_PCR$ncomp){

OOB = c(1:1000)

for (j in 1:1000){

for (m in 1:imp){

data <- complete(tempData, m, include = FALSE)

dataj <- data[index[,j],]

if(m==1) train_boot <- dataj

else train_boot <- rbind.data.frame(train_boot,dataj)

train_boot

}

for (m in 1:imp){

data <- complete(tempData, m, include = FALSE)

dataj <- data[-index[,j],]

if(m==1) validate_boot <- dataj

else validate_boot <- rbind.data.frame(validate_boot,dataj)

validate_boot

}

model_boot <- pcr(log(Dose_mg_week) ~ ., data = train_boot, scale = TRUE)

predicted_dose <- exp(predict (model_boot, validate_boot, ncomp=i))

test_performance <- mean(abs(log(predicted_dose/validate_boot$Dose_mg_week)))

OOB[j] = test_performance

OOB

}

results[i] <- mean(OOB)

results

}

results

plot(results)

#which.min(results) == 8

#Model fit in merged dataset

ncomp_best <- which.min(results)

model_PCR <- pcr(log(Dose_mg_week) ~ ., data = merged, scale = TRUE)

#During prediction in the development dataset, predicted dose is obtained by predicted_dose <- exp(predict(model_PCR, data_dev, ncomp=ncomp_best)). A similar formula is used for the bootstrap/validation datasets.

#Partial least squares regression

#Same as for principal components regression, except that the function plsr() is used instead of pcr().

#k-nearest neighbors

#load package

library(FNN)

#Tuning for k (the number of nearest neighbours)

#Sequence of ks

#k_s <- seq(1, 1090, by = 100)

#k_s <- seq(500, 700, by = 25)

#k_s <- seq(550, 590, by = 10)

k_s <- c(555:570)

results <- c(1:length(k_s))

for (i in 1:length(k_s)){

k <- k_s[i]

OOB = c(1:1000)

for (j in 1:1000){

for (m in 1:imp){

data <- complete(tempData, m, include = FALSE)

dataj <- data[index[,j],]

if(m==1) train_boot <- dataj

else train_boot <- rbind.data.frame(train_boot,dataj)

train_boot

}

train_boot2 <- model.matrix(~ Country_recruitment + Age_years + Weight_kg + Gender + Target_INR + HIV_status + Simvastatin_amiodarone, data=train_boot) #Dummy coding

train_boot2 <- as.data.frame(train_boot2)

train_boot2$`(Intercept)` <- NULL

normalize <- function(x) return ((x - min(x)) / (max(x) - min(x))) #min-max normalization

train_boot_n <- as.data.frame(lapply(train_boot2[1:7], normalize))

train_boot_labels <- log(train_boot$Dose_mg_week) #Labels

for (m in 1:imp){

data <- complete(tempData, m, include = FALSE)

dataj <- data[-index[,j],]

if(m==1) validate_boot <- dataj

else validate_boot <- rbind.data.frame(validate_boot,dataj)

validate_boot

}

validate_boot2 <- model.matrix(~ Country_recruitment + Age_years + Weight_kg + Gender + Target_INR + HIV_status + Simvastatin_amiodarone, data=validate_boot) #Dummy coding

validate_boot2 <- as.data.frame(validate_boot2)

validate_boot2$`(Intercept)` <- NULL

normalize <- function(x) return ((x - min(x)) / (max(x) - min(x))) #min-max normalization

validate_boot_n <- as.data.frame(lapply(validate_boot2[1:7], normalize))

validate_boot_labels <- log(validate_boot$Dose_mg_week) #Labels

predicted_dose <- knn.reg(train_boot_n, validate_boot_n, train_boot_labels, k)$pred

test_performance <- mean(abs(predicted_dose-validate_boot_labels))

OOB[j] = test_performance

OOB

}

results[i] <- mean(OOB)

results

}

results

plot(k_s, results)

#Final model fit in merged dataset

merged2 <- model.matrix(~ Country_recruitment + Age_years + Weight_kg + Gender + Target_INR + HIV_status + Simvastatin_amiodarone, data=merged) #Dummy coding

merged2 <- as.data.frame(merged2)

merged2$`(Intercept)` <- NULL

normalize <- function(x) return ((x - min(x))/(max(x) - min(x))) #min-max normalization

merged_n <- as.data.frame(lapply(merged2[1:7], normalize))

merged_labels <- log(merged$Dose_mg_week) #Labels

k <- 566 #best k

#During prediction in the development dataset, first normalize before obtaining the predicted dose as follows:

data_dev <- complete(tempData, j, include = FALSE)

data_dev2 <- model.matrix(~ Country_recruitment + Age_years + Weight_kg + Gender + Target_INR + HIV_status + Simvastatin_amiodarone, data=data_dev) #Dummy coding

data_dev2 <- as.data.frame(data_dev2)

data_dev2$`(Intercept)` <- NULL

normalize <- function(x) return ((x - min(x))/(max(x) - min(x))) #min-max normalization

data_dev_n <- as.data.frame(lapply(data_dev2[1:7], normalize))

predicted_dose <- exp(knn.reg(merged_n, data_dev_n, merged_labels, k)$pred)

#Similar formulae are used for the bootstrap/validation datasets.

#Artificial neural networks

#load package

library(neuralnet)

#Tuning number of hidden layers. Tested the following combinations individually: c(1), c(2), c(3), c(1,1), c(1,2), c(1,3), c(2,1), c(2,2), c(2,3), c(3,1), c(3,2), c(3,3), c(1,1,1), c(1,1,2), c(1,1,3), c(1,2,1), c(1,2,2), c(1,2,3), c(1,3,1), c(1,3,2), c(1,3,3), c(2,1,1), c(2,1,2), c(2,1,3), c(2,2,1), c(2,2,2), c(2,2,3), c(2,3,1), c(2,3,2), c(2,3,3), c(3,1,1), c(3,1,2), c(3,1,3), c(3,2,1), c(3,2,2), c(3,2,3), c(3,3,1), c(3,3,2), c(3,3,3). c(1) stands for 1 hidden layer, one node; while c(3,1,2) stands for 3 hidden layers with the first, second and third hidden layers having 3, 1, and 2 nodes respectively.

n <- c(1) #or any of the above other combinations.

OOB = c(1:1000)

for (j in 1:1000){

for (m in 1:imp){

data <- complete(tempData, m, include = FALSE)

dataj <- data[index[,j],]

if(m==1) train_boot <- dataj

else train_boot <- rbind.data.frame(train_boot,dataj)

train_boot

}

train_boot2 <- model.matrix(~ Country_recruitment + Age_years + Weight_kg + Gender + Target_INR + HIV_status + Simvastatin_amiodarone, data=train_boot) #Dummy coding

train_boot2 <- as.data.frame(train_boot2)

train_boot2$`(Intercept)` <- NULL

normalize <- function(x) return ((x - min(x))/(max(x) - min(x))) #min-max normalization

train_boot_n <- as.data.frame(lapply(train_boot2[1:7], normalize))

train_boot_n$Dose_mg_week <- train_boot$Dose_mg_week

for (m in 1:imp){

data <- complete(tempData, m, include = FALSE)

dataj <- data[-index[,j],]

if(m==1) validate_boot <- dataj

else validate_boot <- rbind.data.frame(validate_boot,dataj)

validate_boot

}

validate_boot2 <- model.matrix(~ Country_recruitment + Age_years + Weight_kg + Gender + Target_INR + HIV_status + Simvastatin_amiodarone, data=validate_boot) #Dummy coding

validate_boot2 <- as.data.frame(validate_boot2)

validate_boot2$`(Intercept)` <- NULL

normalize <- function(x) return ((x - min(x)) / (max(x) - min(x))) #min-max normalization

validate_boot_n <- as.data.frame(lapply(validate_boot2[1:7], normalize))

validate_boot_n$Dose_mg_week <- validate_boot$Dose_mg_week

tryCatch({

boot_fit <- neuralnet(log(Dose_mg_week) ~., train_boot_n, hidden = n, stepmax=1e6)

predicted_dose <- exp(as.vector(compute(boot_fit, validate_boot_n)$net.result))

test_performance <- mean(abs(log(predicted_dose/validate_boot_n$Dose_mg_week)))

OOB[j] = test_performance

OOB

}, error=function(e){})

}

results <- mean(OOB)

results

write.csv(results, file = paste(paste(n,collapse="_"), ".csv", sep=""))

#Final model fit in merged dataset

merged2 <- model.matrix(~ Country_recruitment + Age_years + Weight_kg + Gender + Target_INR + HIV_status + Simvastatin_amiodarone, data=merged) #Dummy coding

merged2 <- as.data.frame(merged2)

merged2$`(Intercept)` <- NULL

normalize <- function(x) return ((x - min(x))/(max(x) - min(x))) #min-max normalization

merged_n <- as.data.frame(lapply(merged2[1:7], normalize))

merged_n$Dose_mg_week <- merged$Dose_mg_week

n <- c(1)

model_fit <- neuralnet(log(Dose_mg_week) ~., merged_n, hidden = n, stepmax=1e6)

#During prediction in the development dataset, first normalize before obtaining the predicted dose as follows:

data_dev <- complete(tempData, j, include = FALSE)

data_dev2 <- model.matrix(~ Country_recruitment + Age_years + Weight_kg + Gender + Target_INR + HIV_status + Simvastatin_amiodarone, data=data_dev) #Dummy coding

data_dev2 <- as.data.frame(data_dev2)

data_dev2$`(Intercept)` <- NULL

normalize <- function(x) return ((x - min(x))/(max(x) - min(x))) #min-max normalization

data_dev_n <- as.data.frame(lapply(data_dev2[1:7], normalize))

data_dev_n$Dose_mg_week <- data_dev$Dose_mg_week

predicted_dose <- exp(as.vector(compute(model_fit, data_dev_n)$net.result))

#Similar formulae are used for the bootstrap/validation datasets.

#Support vector regression

#load package

library(e1071)

##Linear kernel

#cost_n <- 2^(2:7)

#cost_n <- c(0.001, 0.01, 0.1, 1)

#cost_n <- c(2:5)

cost_n <- seq(1,3,0.1)

epsilon_n <- seq(0,1,0.1)

#epsilon_n <- seq(0,0.4,0.1)

#epsilon_n <- c(0,0.4)

#epsilon_n <- c(0.4)

results_matrix = matrix(0, nrow = (length(epsilon_n)), ncol = (length(cost_n)),

dimnames = list(paste(c(epsilon_n)), paste(c(cost_n))))

for (a in 1:length(cost_n)){

cost_a <- cost_n[a]

results_epsilon <- c(1:length(epsilon_n))

for (b in 1:length(epsilon_n)){

epsilon_b <- epsilon_n[b]

OOB = c(1:1000)

for (j in 1:1000){

for (m in 1:imp){

data <- complete(tempData, m, include = FALSE)

dataj <- data[index[,j],]

if(m==1) train_boot <- dataj

else train_boot <- rbind.data.frame(train_boot,dataj)

train_boot

}

train_boot2 <- model.matrix(~ Country_recruitment + Age_years + Weight_kg + Gender + Target_INR + HIV_status + Simvastatin_amiodarone, data=train_boot) #Dummy coding

train_boot2 <- as.data.frame(train_boot2)

train_boot2$`(Intercept)` <- NULL

normalize <- function(x) return ((x - min(x)) / (max(x) - min(x))) #min-max normalization

train_boot_n <- as.data.frame(lapply(train_boot2[1:7], normalize))

train_boot_n$Dose_mg_week <- train_boot$Dose_mg_week

for (m in 1:imp){

data <- complete(tempData, m, include = FALSE)

dataj <- data[-index[,j],]

if(m==1) validate_boot <- dataj

else validate_boot <- rbind.data.frame(validate_boot,dataj)

validate_boot

}

validate_boot2 <- model.matrix(~ Country_recruitment + Age_years + Weight_kg + Gender + Target_INR + HIV_status + Simvastatin_amiodarone, data=validate_boot) #Dummy coding

validate_boot2 <- as.data.frame(validate_boot2)

validate_boot2$`(Intercept)` <- NULL

normalize <- function(x) return ((x - min(x)) / (max(x) - min(x))) #min-max normalization

validate_boot_n <- as.data.frame(lapply(validate_boot2[1:7], normalize))

validate_boot_n$Dose_mg_week <- validate_boot$Dose_mg_week

tryCatch({

model_fit <- svm(log(Dose_mg_week) ~., train_boot_n, kernel = "linear", scale =FALSE, cost = cost_a, epsilon = epsilon_b)

predicted_dose <- exp(predict(model_fit, validate_boot_n))

test_performance <- mean(abs(log(predicted_dose/validate_boot_n$Dose_mg_week)))

OOB[j] = test_performance

OOB

}, error=function(e){})

}

results_epsilon[b] <- mean(OOB, na.rm=T)

results_epsilon

}

results_matrix[,a] <- results_epsilon

results_matrix

}

results_matrix

write.csv(results_matrix, file = "SVR_linear.csv")

##Radial kernel

#gamma=c(0.1,0.5,1,2,3,4) - Run for each gamma

gamma_n <- 0.1

#cost_n <- 2^(2:7)

#cost_n <- c(0.001, 0.01, 0.1, 1)

#cost_n <- c(2:5)

cost_n <- seq(1,3,0.1)

epsilon_n <- seq(0,1,0.1)

#epsilon_n <- seq(0,0.4,0.1)

#epsilon_n <- c(0,0.4)

#epsilon_n <- c(0.4)

results_matrix = matrix(0, nrow = (length(epsilon_n)), ncol = (length(cost_n)),

dimnames = list(paste(c(epsilon_n)), paste(c(cost_n))))

for (a in 1:length(cost_n)){

cost_a <- cost_n[a]

results_epsilon <- c(1:length(epsilon_n))

for (b in 1:length(epsilon_n)){

epsilon_b <- epsilon_n[b]

OOB = c(1:1000)

for (j in 1:1000){

for (m in 1:imp){

data <- complete(tempData, m, include = FALSE)

dataj <- data[index[,j],]

if(m==1) train_boot <- dataj

else train_boot <- rbind.data.frame(train_boot,dataj)

train_boot

}

train_boot2 <- model.matrix(~ Country_recruitment + Age_years + Weight_kg + Gender + Target_INR + HIV_status + Simvastatin_amiodarone, data=train_boot) #Dummy coding

train_boot2 <- as.data.frame(train_boot2)

train_boot2$`(Intercept)` <- NULL

normalize <- function(x) return ((x - min(x)) / (max(x) - min(x))) #min-max normalization

train_boot_n <- as.data.frame(lapply(train_boot2[1:7], normalize))

train_boot_n$Dose_mg_week <- train_boot$Dose_mg_week

for (m in 1:imp){

data <- complete(tempData, m, include = FALSE)

dataj <- data[-index[,j],]

if(m==1) validate_boot <- dataj

else validate_boot <- rbind.data.frame(validate_boot,dataj)

validate_boot

}

validate_boot2 <- model.matrix(~ Country_recruitment + Age_years + Weight_kg + Gender + Target_INR + HIV_status + Simvastatin_amiodarone, data=validate_boot) #Dummy coding

validate_boot2 <- as.data.frame(validate_boot2)

validate_boot2$`(Intercept)` <- NULL

normalize <- function(x) return ((x - min(x)) / (max(x) - min(x))) #min-max normalization

validate_boot_n <- as.data.frame(lapply(validate_boot2[1:7], normalize))

validate_boot_n$Dose_mg_week <- validate_boot$Dose_mg_week

tryCatch({

model_fit <- svm(log(Dose_mg_week) ~., train_boot_n, kernel = "radial", gamma = gamma_n, scale =FALSE, cost = cost_a, epsilon = epsilon_b)

predicted_dose <- exp(predict(model_fit, validate_boot_n))

test_performance <- mean(abs(log(predicted_dose/validate_boot_n$Dose_mg_week)))

OOB[j] = test_performance

OOB

}, error=function(e){})

}

results_epsilon[b] <- mean(OOB, na.rm=T)

results_epsilon

}

results_matrix[,a] <- results_epsilon

results_matrix

}

results_matrix

write.csv(results_matrix, file = paste(paste(gamma_n,collapse="_"), ".csv", sep=""))

##sigmoid kernel

#gamma=c(0.1,0.5,1) - Run for each gamma as above, with the model fit as below

cost_n <- seq(1,1.5,0.1)

epsilon_n <- seq(0,0.8,0.1)

gamma_n <- 0.1

model_fit <- svm(log(Dose_mg_week) ~., train_boot_n, kernel = "sigmoid", gamma = gamma_n, scale =FALSE, cost = cost_a, epsilon = epsilon_b)

##polynomial kernel

#gamma=c(0.1,0.5,1) - Run for each gamma as above, with the model fit as below

#degree = c(1,2,3) - Also run for each degree

gamma_n <- 0.1

degree_n <- 1

model_fit <- svm(log(Dose_mg_week) ~., train_boot_n, kernel = "polynomial", gamma = gamma_n, degree = degree_n, scale =FALSE, cost = cost_a, epsilon = epsilon_b)

#Final model fit in merged dataset

merged2 <- model.matrix(~ Country_recruitment + Age_years + Weight_kg + Gender + Target_INR + HIV_status + Simvastatin_amiodarone, data=merged) #Dummy coding

merged2 <- as.data.frame(merged2)

merged2$`(Intercept)` <- NULL

normalize <- function(x) return ((x - min(x))/(max(x) - min(x))) #min-max normalization

merged_n <- as.data.frame(lapply(merged2[1:7], normalize))

merged_n$Dose_mg_week <- merged$Dose_mg_week

model_fit <- svm(log(Dose_mg_week) ~., merged_n, kernel = "polynomial", gamma = 0.1, degree = 1, scale =FALSE, cost = 1, epsilon = 0.1)

#During prediction in the development dataset, first normalize before obtaining the predicted dose as follows:

data_dev <- complete(tempData, j, include = FALSE)

data_dev2 <- model.matrix(~ Country_recruitment + Age_years + Weight_kg + Gender + Target_INR + HIV_status + Simvastatin_amiodarone, data=data_dev) #Dummy coding

data_dev2 <- as.data.frame(data_dev2)

data_dev2$`(Intercept)` <- NULL

normalize <- function(x) return ((x - min(x))/(max(x) - min(x))) #min-max normalization

data_dev_n <- as.data.frame(lapply(data_dev2[1:7], normalize))

data_dev_n$Dose_mg_week <- data_dev$Dose_mg_week

predicted_dose <- exp(predict(model_fit, data_dev_n))

#Similar formulae are used for the bootstrap/validation datasets.

#Regression trees

#load package

library(tree)

#Tuning pruning parameter (tree size/number of terminal nodes)

n <- seq(2,14,1)

results <- c(1:length(n))

for (i in 1:length(n)){

prune_n <- n[i]

OOB = c(1:1000)

for (j in 1:1000){

for (m in 1:imp){

data <- complete(tempData, m, include = FALSE)

dataj <- data[index[,j],]

if(m==1) train_boot <- dataj

else train_boot <- rbind.data.frame(train_boot,dataj)

train_boot

}

for (m in 1:imp){

data <- complete(tempData, m, include = FALSE)

dataj <- data[-index[,j],]

if(m==1) validate_boot <- dataj

else validate_boot <- rbind.data.frame(validate_boot,dataj)

validate_boot

}

fit <- tree(log(Dose_mg_week) ~., train_boot)

#plot(fit)

#text(fit ,pretty =0)

model_fit <- prune.tree(fit, best =prune_n)

predicted_dose <- exp(predict(model_fit, validate_boot))

test_performance <- mean(abs(log(predicted_dose/validate_boot$Dose_mg_week)))

OOB[j] = test_performance

OOB

}

results[i] <- mean(OOB)

results

}

results

plot(n, results)

#Final model fit in merged dataset

prune_n <- 4

fit <- tree(log(Dose_mg_week) ~., merged)

model_fit <- prune.tree(fit, best =prune_n)

#Model trees

#load package

library(RWeka)

#Fit model (Tree pruning is incorporated in the model-fitting process)

model_fit <- M5P(log(Dose_mg_week) ~., merged)

#Boosted trees

#load package

library(gbm)

#Tuning

#n.trees_n <- seq(50,500,50), do for each separately

n.trees_n <- 500

n.minobsinnode_n <- c(1,5)

shrinkage_n <- c(0.001, 0.01, 0.1)

results_matrix = matrix(0, nrow = (length(shrinkage_n)), ncol = (length(n.minobsinnode_n)),

dimnames = list(paste(c(shrinkage_n)), paste(c(n.minobsinnode_n))))

for (a in 1:length(n.minobsinnode_n)){

n.minobsinnode_a <- n.minobsinnode_n[a]

results_shrinkage_n <- c(1:length(shrinkage_n))

for (b in 1:length(shrinkage_n)){

shrinkage_b <- shrinkage_n[b]

OOB = c(1:1000)

for (j in 1:1000){

for (m in 1:imp){

data <- complete(tempData, m, include = FALSE)

dataj <- data[index[,j],]

if(m==1) train_boot <- dataj

else train_boot <- rbind.data.frame(train_boot,dataj)

train_boot

}

for (m in 1:imp){

data <- complete(tempData, m, include = FALSE)

dataj <- data[-index[,j],]

if(m==1) validate_boot <- dataj

else validate_boot <- rbind.data.frame(validate_boot,dataj)

validate_boot

}

set.seed (7)

model_fit <- gbm(log(Dose_mg_week) ~., train_boot, distribution="gaussian", n.trees=n.trees_n,

shrinkage=shrinkage_b, n.minobsinnode=n.minobsinnode_a)

predicted_dose <- exp(predict(model_fit, validate_boot))

test_performance <- mean(abs(log(predicted_dose/validate_boot$Dose_mg_week)))

OOB[j] = test_performance

OOB

}

results_shrinkage_n[b] <- mean(OOB)

results_shrinkage_n

}

results_matrix[,a] <- results_shrinkage_n

results_matrix

}

results_matrix

#Final model fit in merged dataset

n.trees_n <- 500

n.minobsinnode_n <- 2

shrinkage_b <- 0.01

model_fit <- gbm(log(Dose_mg_week) ~., merged, distribution="gaussian", n.trees=n.trees_n,

shrinkage=shrinkage_b, n.minobsinnode=n.minobsinnode_n)

#Bagged trees (subset of Random Forest with mtry = p)

#load package

library(randomForest)

#Tuning process similar to regression and boosted trees with ntree <- seq(50,500,50), and nodesize <- c(1:8).

#Final model

model_fit <- randomForest(log(Dose_mg_week) ~., merged, ntree=500, mtry = 7, nodesize = 8)

#Random forest regression

#load package

library(randomForest)

#Tuning process similar to regression and boosted trees with ntree <- seq(50,500,50), nodesize <- c(1:8), and mtry <- c(1:7).

#Final model

model_fit <- randomForest(log(Dose_mg_week) ~., merged, ntree=500, mtry = 1, nodesize = 8)

#Bayesian additive regression trees

#load package

library(bartMachine)

#Tuning number of trees

#nprune_a <- seq(5, 300, by= 50)

#nprune_a <- c(1:5) – did 1 at a time due to time it takes to complete a run

nprune_a <- 1

OOB = c(1:1000)

for (j in 1:1000){

for (m in 1:imp){

data <- complete(tempData, m, include = FALSE)

dataj <- data[index[,j],]

if(m==1) train_boot <- dataj

else train_boot <- rbind.data.frame(train_boot,dataj)

train_boot

}

for (m in 1:imp){

data <- complete(tempData, m, include = FALSE)

dataj <- data[-index[,j],]

if(m==1) validate_boot <- dataj

else validate_boot <- rbind.data.frame(validate_boot,dataj)

validate_boot

}

X <- train_boot[,1:7]

y <- log(train_boot$Dose_mg_week)

X1 <- validate_boot[,1:7]

model_fit <- bartMachine(X,y, num_trees = nprune_a, seed = 7)

predicted_dose <- exp(predict(model_fit, X1))

test_performance <- mean(abs(log(predicted_dose/validate_boot$Dose_mg_week)))

OOB[j] = test_performance

OOB

}

mean(OOB)

#Model fit in merged dataset

nprune_a <- 1

X <- merged[,1:7]

y <- log(merged$Dose_mg_week)

model_fit <- bartMachine(X,y, num_trees = nprune_a, seed = 7)

#During prediction in the development dataset, predicted dose is obtained by predicted_dose <- exp(predict(model_fit, X_dev)), where X_dev <- data_dev[,1:7]. Similar formulae are used for the bootstrap/validation datasets.

#Multivariate adaptive regression splines

#load package

library(earth)

#Tuning number of terms

nprune_a <- (2:11)

results <- c(1:length(nprune_a))

for (i in 1:length(nprune_a)){

OOB = c(1:1000)

for (j in 1:1000){

for (m in 1:imp){

data <- complete(tempData, m, include = FALSE)

dataj <- data[index[,j],]

if(m==1) train_boot <- dataj

else train_boot <- rbind.data.frame(train_boot,dataj)

train_boot

}

for (m in 1:imp){

data <- complete(tempData, m, include = FALSE)

dataj <- data[-index[,j],]

if(m==1) validate_boot <- dataj

else validate_boot <- rbind.data.frame(validate_boot,dataj)

validate_boot

}

X <- model.matrix(Dose_mg_week ~ ., train_boot)[, -1]

y <- log(train_boot$Dose_mg_week)

X1 <- model.matrix(Dose_mg_week ~ ., validate_boot)[, -1]

model_fit <- earth(X,y, pmethod = "exhaustive", nprune = nprune_a[i])

predicted_dose <- exp(predict(model_fit, X1))

test_performance <- mean(abs(log(predicted_dose/validate_boot$Dose_mg_week)))

OOB[j] = test_performance

OOB

}

results[i] <- mean(OOB)

results

}

results

plot(nprune_a, results)

#Model fit in merged dataset

nprune_a <- 4

X <- model.matrix(Dose_mg_week ~ ., merged)[, -1]

y <- log(merged$Dose_mg_week)

model_fit <- earth(X,y, pmethod = "exhaustive", nprune = nprune_a)

#During prediction in the development dataset, predicted dose is obtained by predicted_dose <- exp(predict(model_fit, X_dev)), where X_dev <- model.matrix(Dose_mg_week ~ ., data_dev)[, -1]. Similar formulae are used for the bootstrap/validation datasets.

#Cubist

#load package

library(Cubist)

#Tuning committees

committees_a <- seq(10,100,10)

results <- c(1:length(committees_a))

for (i in 1:length(committees_a)){

OOB = c(1:1000)

for (j in 1:1000){

for (m in 1:imp){

data <- complete(tempData, m, include = FALSE)

dataj <- data[index[,j],]

if(m==1) train_boot <- dataj

else train_boot <- rbind.data.frame(train_boot,dataj)

train_boot

}

for (m in 1:imp){

data <- complete(tempData, m, include = FALSE)

dataj <- data[-index[,j],]

if(m==1) validate_boot <- dataj

else validate_boot <- rbind.data.frame(validate_boot,dataj)

validate_boot

}

X <- model.matrix(Dose_mg_week ~ ., train_boot)[, -1]

y <- log(train_boot$Dose_mg_week)

X1 <- model.matrix(Dose_mg_week ~ ., validate_boot)[, -1]

model_fit <- cubist(X,y, committees = committees_a[i])

predicted_dose <- exp(predict(model_fit, X1))

test_performance <- mean(abs(log(predicted_dose/validate_boot$Dose_mg_week)))

OOB[j] = test_performance

OOB

}

results[i] <- mean(OOB)

results

}

results

plot(committees_a, results)

#Model fit in merged dataset

X <- model.matrix(Dose_mg_week ~ ., merged)[, -1]

y <- log(merged$Dose_mg_week)

model_fit <- cubist(X,y, committees = 100)

#During prediction in the development dataset, predicted dose is obtained by predicted_dose <- exp(predict(model_fit, X_dev)), where X_dev <- model.matrix(Dose_mg_week ~ ., data_dev)[, -1]. Similar formulae are used for the bootstrap/validation datasets.

#eXtreme Gradient Boosting

#load package

library(xgboost)

#Tuning

#General parameters (booster = gbtree, gblinear, dart)

#Tree booster (gbtree or dart)

#eta/learning rate (Step size shrinkage used in update to prevents overfitting, default = 0.3), eta = c(0, 0.2, 0.4, 0.6, 0.8, 1)

#gamma [default=0, alias: min_split_loss, range: [0,???]], gamma = 0, 10, 100

#max_depth [default=6], max_depth = c(2,4,6,8)

#nrounds = c(5, 10, 100)

##gbtree

booster_n = 'gbtree' #also tried 'dart'

nrounds_n = 5 #tried others shown above

gamma_n = 0 #tried others shown above

max_depth_n = c(2,4,6,8)

eta_n = c(0, 0.2, 0.4, 0.6, 0.8, 1)

results_matrix = matrix(0, nrow = (length(eta_n)), ncol = (length(max_depth_n)),

dimnames = list(paste(c(eta_n)), paste(c(max_depth_n))))

for (a in 1:length(max_depth_n)){

max_depth_n_a <- max_depth_n[a]

results_eta_n <- c(1:length(eta_n))

for (b in 1:length(eta_n)){

eta_n_b <- eta_n[b]

OOB = c(1:1000)

for (j in 1:1000){

for (m in 1:imp){

data <- complete(tempData, m, include = FALSE)

dataj <- data[index[,j],]

if(m==1) train_boot <- dataj

else train_boot <- rbind.data.frame(train_boot,dataj)

train_boot

}

for (m in 1:imp){

data <- complete(tempData, m, include = FALSE)

dataj <- data[-index[,j],]

if(m==1) validate_boot <- dataj

else validate_boot <- rbind.data.frame(validate_boot,dataj)

validate_boot

}

X = model.matrix(Dose_mg_week ~ ., train_boot)[, -1]

y = log(train_boot$Dose_mg_week)

model_fit <- xgboost(data = X, label = y, booster = booster_n, eta = eta_n_b, gamma = gamma_n, max_depth = max_depth_n_a, nrounds = nrounds_n)

X2 = model.matrix(Dose_mg_week ~ ., validate_boot)[, -1]

predicted_dose <- exp(predict(model_fit, X2))

test_performance <- mean(abs(log(predicted_dose/validate_boot$Dose_mg_week)))

OOB[j] = test_performance

OOB

}

results_eta_n[b] <- mean(OOB)

results_eta_n

}

results_matrix[,a] <- results_eta_n

results_matrix

}

results_matrix

#If the booster object is DART type, predict() will perform dropouts, i.e. only some of the trees will be evaluated. This will produce incorrect results if data is not the training data. To obtain correct results on test sets, set ntree_limit to a nonzero value

##Linear booster

#lambda [default=0, alias: reg_lambda], lambda = c(0, 1, 2)

#alpha [default=0, alias: reg_alpha], alpha = c(0, 1, 2)

#updater [default= shotgun], updater = c(shotgun, coord_descent)

#nrounds = c(5, 10, 100)

booster_n = 'gblinear'

nrounds_n = 5

updater_n = 'shotgun'

lambda_n = c(0, 1, 2)

alpha_n = c(0, 1, 2)

results_matrix = matrix(0, nrow = (length(alpha_n)), ncol = (length(lambda_n)),

dimnames = list(paste(c(alpha_n)), paste(c(lambda_n))))

for (a in 1:length(lambda_n)){

lambda_n_a <- lambda_n[a]

results_alpha_n <- c(1:length(alpha_n))

for (b in 1:length(alpha_n)){

alpha_n_b <- alpha_n[b]

OOB = c(1:1000)

for (j in 1:1000){

for (m in 1:imp){

data <- complete(tempData, m, include = FALSE)

dataj <- data[index[,j],]

if(m==1) train_boot <- dataj

else train_boot <- rbind.data.frame(train_boot,dataj)

train_boot

}

for (m in 1:imp){

data <- complete(tempData, m, include = FALSE)

dataj <- data[-index[,j],]

if(m==1) validate_boot <- dataj

else validate_boot <- rbind.data.frame(validate_boot,dataj)

validate_boot

}

X = model.matrix(Dose_mg_week ~ ., train_boot)[, -1]

y = log(train_boot$Dose_mg_week)

model_fit <- xgboost(data = X, label = y, booster = booster_n, alpha = alpha_n_b, updater = updater_n, lambda = lambda_n_a, nrounds = nrounds_n)

X2 = model.matrix(Dose_mg_week ~ ., validate_boot)[, -1]

predicted_dose <- exp(predict(model_fit, X2))

test_performance <- mean(abs(log(predicted_dose/validate_boot$Dose_mg_week)))

OOB[j] = test_performance

OOB

}

results_alpha_n[b] <- mean(OOB)

results_alpha_n

}

results_matrix[,a] <- results_alpha_n

results_matrix

}

results_matrix

#Final model fit in merged dataset

X = model.matrix(Dose_mg_week ~ ., merged)[, -1]

y = log(merged$Dose_mg_week)

booster_n = 'gbtree'

nrounds_n = 100

gamma_n = 0

max_depth_n = 1

eta_n = 0.1

model_fit <- xgboost(data = X, label = y, booster = booster_n, eta = eta_n, gamma = gamma_n, max_depth = max_depth_n, nrounds = nrounds_n)

#During prediction in the development dataset, predicted dose is obtained by predicted_dose <- exp(predict(model_fit, X_dev)), where X_dev <- model.matrix(Dose_mg_week ~ ., data_dev)[, -1]. Similar formulae are used for the bootstrap/validation datasets.

**##Example dataset**

This dataset was derived from the publicly available de-identified International Warfarin Pharmacogenetics Consortium (IWPC) IWPC ethnicity dataset (available from <https://www.pharmgkb.org/downloads>, under the sub-heading “International Warfarin Pharmacogenetics Consortium (IWPC)”). A random selection of 364 blacks was obtained, and Country of recruitment/HIV-status randomly added in proportions similar to those of the War-PATH dataset. Finally, weight data was removed from some patients so that 11 (3%) cases were missing weight. This dataset is strictly for replicating the key modeling and simulation steps described above, with results provided below. To replicate the results reported in the main manuscript, the War-PATH datasets are available from the corresponding author on reasonable request.

| **Country_recruitment** | **Age_years** | **Weight_kg** | **Gender** | **Simvastatin_amiodarone** | **Target_INR** | **HIV_status** | **Dose_mg_week** |
| --- | --- | --- | --- | --- | --- | --- | --- |
| South Africa | 40 | 45 | Female | No | "3.0" | Negative | 30 |
| Uganda | 70 | 45 | Female | No | "2.5" | Negative | 17.5 |
| South Africa | 70 | 46.04 | Female | No | "3.0" | Negative | 36 |
| South Africa | 50 | 48 | Female | No | "3.0" | Negative | 35 |
| Uganda | 80 | 49 | Female | No | "2.5" | Negative | 32 |
| South Africa | 20 | 49.1 | Female | No | "2.5" | Negative | 35 |
| South Africa | 50 | 50 | Female | No | "3.0" | Positive | 27.5 |
| South Africa | 40 | 50 | Female | No | "2.5" | Negative | 22.5 |
| South Africa | 30 | 51 | Female | No | "2.5" | Negative | 42.5 |
| Uganda | 50 | 51.4 | Female | No | "2.5" | Positive | 30 |
| South Africa | 40 | 53.2 | Female | No | "2.5" | Negative | 52.5 |
| South Africa | 70 | 53.52 | Male | No | "2.5" | Negative | 20 |
| Uganda | 40 | 54 | Female | No | "2.5" | Positive | 42 |
| Uganda | 30 | 54.43 | Female | No | "2.5" | Unknown | 67 |
| South Africa | 40 | 54.5 | Female | No | "2.5" | Negative | 38 |
| South Africa | 50 | 55 | Female | No | "3.0" | Positive | 32.5 |
| South Africa | 40 | 55.5 | Male | No | "2.5" | Negative | 35 |
| South Africa | 60 | 56 | Female | No | "3.0" | Positive | 45 |
| South Africa | 20 | 56.2 | Male | No | "2.5" | Unknown | 35 |
| Uganda | 40 | 56.9 | Female | No | "3.0" | Negative | 72.5 |
| Uganda | 60 | 57.3 | Female | No | "2.5" | Negative | 30 |
| South Africa | 40 | 57.7 | Female | No | "2.5" | Unknown | 29.17 |
| Uganda | 30 | 58 | Female | No | "3.0" | Negative | 32.5 |
| South Africa | 70 | 58.51 | Female | No | "2.5" | Negative | 14 |
| South Africa | 50 | 58.6 | Male | No | "2.5" | Negative | 47.53 |
| South Africa | 70 | 59.87 | Male | No | "2.5" | Negative | 16 |
| South Africa | 40 | 61.4 | Female | No | "2.5" | Negative | 35 |
| South Africa | 40 | 61.6 | Female | No | "2.5" | Negative | 35 |
| South Africa | 70 | 62 | Male | No | "2.5" | Negative | 14 |
| South Africa | 60 | 62.6 | Male | Yes | "2.5" | Negative | 12.53 |
| South Africa | 80 | 62.7 | Female | No | "2.5" | Unknown | 25 |
| South Africa | 40 | 63 | Female | No | "3.0" | Unknown | 56 |
| Uganda | 90 | 63.05 | Female | No | "2.5" | Unknown | 26 |
| South Africa | 70 | 63.5 | Male | No | "2.5" | Negative | 32.5 |
| South Africa | 70 | 63.5 | Male | No | "2.5" | Negative | 25 |
| South Africa | 80 | 63.6 | Female | No | "2.5" | Negative | 24.99 |
| Uganda | 80 | 63.6 | Male | No | "2.5" | Negative | 52.5 |
| South Africa | 50 | 63.6 | Female | No | "2.5" | Negative | 30 |
| Uganda | 20 | 64 | Female | No | "2.5" | Unknown | 42.5 |
| South Africa | 50 | 64.1 | Female | No | "2.5" | Positive | 30 |
| Uganda | 80 | 64.2 | Male | No | "2.5" | Negative | 24.17 |
| South Africa | 20 | 64.5 | Male | No | "2.5" | Negative | 60 |
| South Africa | 50 | 64.5 | Female | No | "2.5" | Positive | 55 |
| Uganda | 40 | 64.86 | Male | No | "2.5" | Negative | 70 |
| South Africa | 40 | 65 | Female | No | "3.0" | Positive | 35 |
| South Africa | 60 | 65 | Female | No | "2.5" | Negative | 45 |
| South Africa | 50 | 65.32 | Female | No | "3.0" | Negative | 49 |
| Uganda | 60 | 65.8 | Male | No | "3.0" | Negative | 28 |
| South Africa | 80 | 65.8 | Male | No | "2.5" | Negative | 42 |
| Uganda | 70 | 66.68 | Female | No | "2.5" | Negative | 14 |
| South Africa | 50 | 66.9 | Male | No | "2.5" | Negative | 28 |
| Uganda | 60 | 67 | Male | No | "2.5" | Unknown | 35 |
| Uganda | 50 | 67.1 | Female | No | "2.5" | Negative | 35 |
| South Africa | 50 | 67.13 | Male | No | "2.5" | Negative | 53 |
| Uganda | 80 | 67.3 | Female | Yes | "2.5" | Negative | 22 |
| South Africa | 40 | 67.3 | Male | No | "2.5" | Negative | 32.5 |
| Uganda | 50 | 67.59 | Female | No | "2.5" | Positive | 43 |
| South Africa | 80 | 68 | Female | No | "2.5" | Negative | 30 |
| South Africa | 80 | 68 | Female | No | "2.5" | Negative | 30 |
| Uganda | 30 | 68 | Male | Yes | "2.5" | Negative | 35 |
| Uganda | 50 | 68 | Female | No | "3.0" | Negative | 27.5 |
| South Africa | 50 | 68 | Female | No | "2.5" | Negative | 30 |
| South Africa | 18 | 68 | Female | No | "2.5" | Negative | 14 |
| Uganda | 80 | 68.04 | Female | No | "2.5" | Negative | 21 |
| Uganda | 90 | 68.2 | Female | No | "2.5" | Positive | 40 |
| Uganda | 70 | 68.2 | Male | No | "2.5" | Negative | 30 |
| South Africa | 20 | 68.2 | Female | No | "2.5" | Unknown | 24.99 |
| South Africa | 30 | 68.2 | Female | No | "2.5" | Negative | 17.5 |
| Uganda | 60 | 68.6 | Male | No | "3.0" | Negative | 52.5 |
| Uganda | 60 | 68.6 | Female | No | "2.5" | Positive | 27.51 |
| South Africa | 80 | 68.95 | Male | No | "2.5" | Negative | 42 |
| South Africa | 50 | 69.1 | Female | No | "2.5" | Negative | 36 |
| Uganda | 30 | 69.1 | Male | No | "2.5" | Positive | 17.5 |
| Uganda | 70 | 70 | Female | No | "2.5" | Negative | 30 |
| South Africa | 70 | 70 | Male | No | "2.5" | Negative | 27.5 |
| Uganda | 50 | 70 | Female | No | "2.5" | Negative | 40 |
| Uganda | 40 | 70 | Female | No | "2.5" | Negative | 35 |
| South Africa | 80 | 70 | Female | No | "2.5" | Negative | 30 |
| Uganda | 60 | 70.3 | Female | No | "2.5" | Negative | 42 |
| Uganda | 70 | 70.31 | Male | No | "2.5" | Negative | 56 |
| South Africa | 40 | 70.5 | Female | No | "2.5" | Negative | 45 |
| Uganda | 70 | 70.76 | Male | No | "2.5" | Positive | 58 |
| Uganda | 70 | 71 | Female | No | "2.5" | Negative | 40 |
| South Africa | 60 | 71.4 | Female | No | "2.5" | Negative | 32 |
| Uganda | 60 | 71.8 | Male | No | "2.5" | Negative | 60 |
| South Africa | 80 | 72.3 | Male | No | "2.5" | Negative | 27.51 |
| Uganda | 60 | 72.3 | Male | No | "2.5" | Negative | 75 |
| South Africa | 70 | 72.3 | Female | Yes | "2.5" | Negative | 27.02 |
| South Africa | 80 | 72.3 | Female | No | "2.5" | Negative | 35 |
| Uganda | 60 | 72.57 | Male | No | "2.5" | Negative | 42 |
| Uganda | 60 | 72.7 | Female | No | "2.5" | Negative | 22 |
| Uganda | 60 | 73 | Female | No | "2.5" | Negative | 35 |
| South Africa | 50 | 73 | Male | No | "3.0" | Negative | 37.5 |
| South Africa | 70 | 73 | Female | No | "2.5" | Negative | 35 |
| South Africa | 50 | 73.6 | Female | No | "2.5" | Negative | 10 |
| South Africa | 80 | 73.6 | Male | No | "2.5" | Positive | 22.5 |
| Uganda | 40 | 74.5 | Female | No | "2.5" | Positive | 21 |
| South Africa | 60 | 75 | Female | No | "2.5" | Negative | 42.5 |
| Uganda | 50 | 75 | Female | No | "2.5" | Negative | 25 |
| South Africa | 80 | 75 | Male | No | "2.5" | Negative | 30 |
| South Africa | 30 | 75 | Female | No | "2.5" | Positive | 30 |
| South Africa | 50 | 75 | Male | No | "2.5" | Negative | 17.5 |
| South Africa | 70 | 75.3 | Female | No | "2.5" | Negative | 28 |
| Uganda | 60 | 75.5 | Female | No | "2.5" | Negative | 52.5 |
| South Africa | 50 | 76 | Female | Yes | "2.5" | Unknown | 45.67 |
| Uganda | 70 | 76.4 | Male | No | "2.5" | Negative | 30 |
| Uganda | 70 | 76.4 | Female | No | "2.5" | Negative | 24.99 |
| South Africa | 40 | 76.8 | Female | No | "3.0" | Negative | 45.38 |
| Uganda | 40 | 77 | Female | No | "2.5" | Negative | 25 |
| Uganda | 50 | 77 | Female | No | "2.5" | Negative | 40 |
| South Africa | 80 | 77.11 | Female | No | "2.5" | Negative | 28 |
| South Africa | 40 | 77.11 | Female | No | "2.5" | Negative | 32 |
| Uganda | 40 | 77.3 | Female | No | "2.5" | Negative | 17.99 |
| Uganda | 60 | 77.3 | Female | No | "2.5" | Negative | 33.75 |
| South Africa | 40 | 77.3 | Female | No | "2.5" | Negative | 20.02 |
| Uganda | 70 | 77.3 | Female | No | "2.5" | Negative | 44 |
| South Africa | 60 | 77.3 | Female | No | "2.5" | Negative | 44.03 |
| Uganda | 40 | 77.3 | Male | No | "2.5" | Negative | 45 |
| Uganda | 40 | 77.6 | Female | No | "2.5" | Negative | 30.52 |
| Uganda | 70 | 77.7 | Female | No | "2.5" | Negative | 22.5 |
| South Africa | 60 | 78 | Female | No | "2.5" | Negative | 30 |
| South Africa | 60 | 78 | Female | No | "2.5" | Negative | 35 |
| Uganda | 50 | 78 | Female | No | "3.0" | Negative | 52.5 |
| South Africa | 70 | 78 | Female | No | "2.5" | Unknown | 32.5 |
| South Africa | 70 | 78.02 | Female | No | "2.5" | Positive | 55 |
| South Africa | 50 | 78.2 | Male | No | "2.5" | Negative | 35 |
| Uganda | 60 | 78.2 | Female | No | "2.5" | Positive | 35 |
| South Africa | 70 | 78.2 | Female | No | "2.5" | Negative | 26.25 |
| South Africa | 50 | 78.47 | Female | No | "2.5" | Negative | 35 |
| Uganda | 50 | 79 | Female | No | "2.5" | Negative | 19 |
| Uganda | 70 | 79.5 | Male | No | "2.5" | Negative | 40 |
| Uganda | 80 | 79.5 | Female | No | "2.5" | Negative | 50 |
| South Africa | 50 | 79.55 | Female | No | "2.5" | Positive | 55 |
| South Africa | 80 | 79.6 | Male | No | "2.5" | Negative | 55.02 |
| South Africa | 40 | 79.8 | Male | No | "2.5" | Unknown | 32.5 |
| Uganda | 50 | 80 | Female | No | "2.5" | Negative | 32.5 |
| Uganda | 60 | 80 | Female | No | "2.5" | Negative | 25 |
| South Africa | 50 | 80 | Male | Yes | "2.5" | Negative | 52.5 |
| South Africa | 20 | 80.06 | Female | No | "2.5" | Negative | 42 |
| Uganda | 40 | 80.9 | Female | No | "2.5" | Negative | 96.39 |
| South Africa | 50 | 81 | Female | No | "3.0" | Positive | 12.5 |
| Uganda | 50 | 81.4 | Male | No | "2.5" | Unknown | 35 |
| South Africa | 40 | 81.4 | Male | No | "2.5" | Negative | 10 |
| South Africa | 40 | 81.4 | Female | No | "2.5" | Negative | 85 |
| South Africa | 30 | 81.8 | Female | No | "2.5" | Negative | 52.5 |
| South Africa | 40 | 81.8 | Male | No | "2.5" | Positive | 52.5 |
| South Africa | 50 | 81.8 | Male | No | "2.5" | Negative | 45 |
| Uganda | 70 | 81.8 | Female | No | "2.5" | Negative | 21 |
| South Africa | 70 | 81.8 | Male | No | "2.5" | Unknown | 42 |
| Uganda | 70 | 81.8 | Female | No | "2.5" | Positive | 14 |
| South Africa | 60 | 82 | Male | No | "3.0" | Negative | 35 |
| Uganda | 60 | 82 | Female | No | "2.5" | Negative | 45 |
| South Africa | 70 | 82.3 | Female | No | "2.5" | Negative | 32.48 |
| South Africa | 50 | 82.4 | Male | No | "2.5" | Negative | 35 |
| South Africa | 70 | 82.55 | Male | No | "2.5" | Negative | 35 |
| Uganda | 50 | 82.6 | Male | No | "2.5" | Negative | 37.5 |
| Uganda | 80 | 82.7 | Male | No | "2.5" | Negative | 13.75 |
| Uganda | 50 | 82.9 | Female | No | "2.5" | Negative | 28 |
| Uganda | 40 | 83 | Female | No | "3.0" | Negative | 15 |
| Uganda | 60 | 83.01 | Female | No | "2.5" | Negative | 35 |
| Uganda | 50 | 83.2 | Male | No | "2.5" | Negative | 22.5 |
| Uganda | 30 | 83.9 | Female | No | "2.5" | Negative | 84 |
| Uganda | 70 | 83.91 | Male | No | "3.0" | Positive | 56 |
| South Africa | 70 | 84 | Female | No | "2.5" | Negative | 28 |
| South Africa | 70 | 84.1 | Male | No | "2.5" | Negative | 37.52 |
| Uganda | 60 | 84.1 | Male | Yes | "2.5" | Negative | 35 |
| Uganda | 70 | 84.1 | Male | No | "2.5" | Negative | 24.99 |
| South Africa | 70 | 84.5 | Male | No | "2.5" | Negative | 47.5 |
| South Africa | 20 | 85 | Male | No | "3.0" | Negative | 60 |
| Uganda | 70 | 85 | Female | No | "2.5" | Negative | 24.99 |
| South Africa | 60 | 85.1 | Female | No | "2.5" | Positive | 24.22 |
| Uganda | 60 | 85.9 | Female | No | "2.5" | Positive | 32 |
| Uganda | 60 | 85.9 | Male | No | "2.5" | Negative | 26.32 |
| South Africa | 40 | 85.9 | Male | No | "2.5" | Negative | 42.5 |
| Uganda | 50 | 86 | Female | No | "2.5" | Positive | 47.5 |
| South Africa | 40 | 86 | Male | No | "2.5" | Negative | 87.5 |
| Uganda | 70 | 86.1 | Male | Yes | "2.5" | Unknown | 21.63 |
| South Africa | 70 | 86.4 | Female | No | "2.5" | Negative | 17.99 |
| South Africa | 50 | 87 | Female | No | "2.5" | Positive | 27.5 |
| South Africa | 70 | 87.09 | Female | No | "2.5" | Negative | 55 |
| Uganda | 50 | 87.1 | Male | No | "2.5" | Positive | 46 |
| Uganda | 70 | 87.3 | Male | No | "2.5" | Negative | 20 |
| Uganda | 60 | 87.3 | Female | No | "2.5" | Negative | 48 |
| Uganda | 50 | 87.7 | Female | No | "2.5" | Unknown | 60 |
| South Africa | 70 | 88.2 | Male | No | "2.5" | Negative | 22.5 |
| Uganda | 70 | 88.4 | Female | No | "2.5" | Negative | 21 |
| Uganda | 80 | 88.45 | Male | No | "2.5" | Negative | 24 |
| Uganda | 50 | 88.45 | Female | No | "3.0" | Negative | 34 |
| Uganda | 50 | 88.6 | Female | No | "2.5" | Negative | 42 |
| Uganda | 30 | 88.6 | Female | No | "2.5" | Negative | 56.25 |
| Uganda | 60 | 88.6 | Male | No | "2.5" | Negative | 32.5 |
| South Africa | 60 | 88.9 | Female | No | "2.5" | Negative | 27.5 |
| South Africa | 50 | 89 | Male | No | "2.5" | Negative | 80 |
| South Africa | 60 | 89.1 | Male | No | "2.5" | Negative | 35 |
| Uganda | 70 | 89.1 | Male | No | "2.5" | Positive | 10.5 |
| South Africa | 40 | 90 | Female | No | "3.0" | Positive | 42 |
| Uganda | 50 | 90 | Male | No | "3.0" | Negative | 28 |
| South Africa | 50 | 90 | Female | No | "2.5" | Negative | 40 |
| Uganda | 50 | 90 | Female | No | "3.0" | Negative | 40 |
| South Africa | 40 | 90.1 | Male | No | "2.5" | Negative | 55 |
| South Africa | 70 | 90.5 | Female | No | "2.5" | Negative | 60 |
| South Africa | 40 | 90.7 | Male | No | "2.5" | Negative | 37.5 |
| Uganda | 60 | 90.7 | Female | No | "2.5" | Negative | 25 |
| South Africa | 50 | 90.9 | Female | No | "2.5" | Negative | 12.5 |
| South Africa | 60 | 90.9 | Male | No | "2.5" | Negative | 27.51 |
| Uganda | 80 | 90.9 | Female | No | "2.5" | Negative | 35 |
| South Africa | 50 | 90.9 | Male | No | "2.5" | Negative | 49.98 |
| South Africa | 40 | 90.9 | Male | No | "2.5" | Negative | 52.5 |
| Uganda | 70 | 90.91 | Female | Yes | "2.5" | Negative | 30 |
| Uganda | 60 | 91 | Male | No | "2.5" | Negative | 42.5 |
| South Africa | 80 | 91 | Female | Yes | "2.5" | Negative | 42.5 |
| South Africa | 70 | 91.5 | Female | No | "2.5" | Positive | 39.9 |
| Uganda | 40 | 91.8 | Female | No | "2.5" | Negative | 56.25 |
| Uganda | 50 | 92 | Female | No | "3.0" | Negative | 37.5 |
| South Africa | 70 | 92.3 | Female | No | "2.5" | Positive | 49.98 |
| South Africa | 30 | 92.5 | Female | No | "2.5" | Positive | 35 |
| South Africa | 50 | 92.5 | Female | No | "2.5" | Negative | 126 |
| Uganda | 70 | 93 | Female | No | "2.5" | Unknown | 42 |
| Uganda | 60 | 93.2 | Female | No | "2.5" | Negative | 35 |
| South Africa | 70 | 93.64 | Male | No | "2.5" | Positive | 42.5 |
| Uganda | 50 | 93.89 | Male | No | "2.5" | Negative | 43 |
| South Africa | 50 | 94.1 | Female | No | "2.5" | Negative | 52.5 |
| South Africa | 40 | 95 | Female | No | "2.5" | Negative | 35 |
| Uganda | 70 | 95 | Female | No | "2.5" | Positive | 37.5 |
| Uganda | 50 | 95 | Female | No | "2.5" | Negative | 33 |
| South Africa | 60 | 95 | Female | No | "2.5" | Negative | 71.25 |
| Uganda | 50 | 95 | Female | Yes | "2.5" | Negative | 35 |
| Uganda | 30 | 95.2 | Female | No | "3.0" | Positive | 70 |
| South Africa | 40 | 95.5 | Female | No | "2.5" | Negative | 45.01 |
| Uganda | 60 | 95.5 | Male | No | "2.5" | Negative | 42.49 |
| South Africa | 50 | 95.5 | Male | No | "2.5" | Negative | 42.5 |
| Uganda | 60 | 95.7 | Male | No | "2.5" | Negative | 50 |
| South Africa | 60 | 95.9 | Male | No | "2.5" | Negative | 47.5 |
| South Africa | 60 | 95.91 | Female | No | "2.5" | Positive | 70 |
| Uganda | 80 | 97 | Male | No | "2.5" | Negative | 43.68 |
| Uganda | 50 | 97.7 | Male | No | "2.5" | Negative | 72.5 |
| South Africa | 70 | 98 | Female | No | "2.5" | Positive | 40 |
| South Africa | 70 | 98.6 | Male | No | "2.5" | Negative | 50 |
| Uganda | 60 | 98.9 | Male | No | "2.5" | Positive | 35 |
| South Africa | 60 | 99.7 | Male | No | "2.5" | Negative | 35 |
| South Africa | 40 | 99.79 | Male | No | "2.5" | Negative | 40 |
| Uganda | 50 | 99.79 | Female | No | "2.5" | Negative | 18 |
| South Africa | 50 | 99.8 | Female | No | "2.5" | Negative | 52 |
| Uganda | 80 | 100 | Female | No | "2.5" | Unknown | 42 |
| South Africa | 30 | 100 | Male | No | "2.5" | Negative | 59.99 |
| South Africa | 50 | 100 | Male | No | "2.5" | Negative | 62.5 |
| South Africa | 40 | 100 | Male | No | "2.5" | Negative | 28 |
| South Africa | 50 | 100 | Male | No | "2.5" | Unknown | 60 |
| South Africa | 40 | 100 | Male | No | "2.5" | Negative | 70 |
| Uganda | 50 | 100 | Male | No | "2.5" | Negative | 55 |
| South Africa | 60 | 100 | Female | No | "2.5" | Negative | 21.25 |
| South Africa | 70 | 100 | Male | No | "2.5" | Negative | 60 |
| Uganda | 40 | 100 | Female | No | "2.5" | Negative | 40 |
| Uganda | 70 | 100.9 | Male | Yes | "2.5" | Negative | 22 |
| South Africa | 50 | 102 | Male | Yes | "2.5" | Negative | 35 |
| Uganda | 50 | 102.3 | Male | No | "2.5" | Negative | 45.01 |
| South Africa | 40 | 102.3 | Male | No | "2.5" | Positive | 32.5 |
| Uganda | 50 | 102.7 | Female | No | "2.5" | Negative | 70 |
| Uganda | 60 | 102.7 | Female | No | "2.5" | Positive | 38 |
| South Africa | 60 | 102.7 | Male | No | "2.5" | Negative | 70 |
| South Africa | 60 | 102.97 | Female | No | "2.5" | Negative | 40 |
| Uganda | 60 | 103 | Female | No | "2.5" | Positive | 31.94 |
| South Africa | 20 | 103.2 | Female | No | "2.5" | Negative | 35 |
| Uganda | 50 | 104.33 | Female | No | "2.5" | Unknown | 53 |
| South Africa | 50 | 104.5 | Female | No | "2.5" | Negative | 52 |
| Uganda | 70 | 104.5 | Male | No | "2.5" | Positive | 80 |
| South Africa | 40 | 104.5 | Female | No | "2.5" | Negative | 36 |
| Uganda | 50 | 105.5 | Male | No | "2.5" | Negative | 45 |
| South Africa | 60 | 106 | Female | No | "3.0" | Negative | 38.5 |
| South Africa | 50 | 106.14 | Male | No | "2.5" | Negative | 54 |
| South Africa | 40 | 106.59 | Male | No | "3.0" | Negative | 67.5 |
| South Africa | 60 | 106.8 | Female | No | "2.5" | Negative | 32.5 |
| Uganda | 50 | 106.8 | Female | No | "2.5" | Negative | 25 |
| Uganda | 60 | 107.05 | Female | No | "2.5" | Negative | 28 |
| South Africa | 30 | 107.3 | Male | Yes | "2.5" | Negative | 42 |
| South Africa | 50 | 108 | Male | No | "2.5" | Negative | 30 |
| South Africa | 60 | 108 | Male | No | "2.5" | Positive | 30 |
| South Africa | 30 | 108 | Male | No | "2.5" | Negative | 37.5 |
| Uganda | 60 | 108.4 | Male | No | "3.0" | Positive | 60 |
| South Africa | 30 | 108.6 | Female | No | "2.5" | Negative | 62.5 |
| South Africa | 70 | 108.9 | Female | No | "2.5" | Negative | 49 |
| Uganda | 40 | 109 | Female | No | "2.5" | Positive | 72 |
| Uganda | 60 | 109.1 | Male | No | "2.5" | Positive | 35 |
| Uganda | 60 | 109.1 | Female | No | "2.5" | Negative | 26.74 |
| Uganda | 50 | 110 | Male | Yes | "2.5" | Positive | 35 |
| South Africa | 60 | 110 | Male | No | "2.5" | Negative | 22.5 |
| South Africa | 60 | 110.6 | Female | No | "2.5" | Negative | 35 |
| South Africa | 50 | 111.36 | Female | No | "2.5" | Negative | 62.5 |
| Uganda | 70 | 112.3 | Female | No | "2.5" | Negative | 37.5 |
| South Africa | 30 | 112.49 | Male | No | "2.5" | Negative | 49 |
| Uganda | 50 | 112.5 | Female | No | "2.5" | Negative | 42.5 |
| South Africa | 50 | 113 | Female | No | "2.5" | Negative | 52.5 |
| Uganda | 70 | 113 | Male | No | "2.5" | Negative | 46.9 |
| Uganda | 40 | 113.2 | Female | No | "2.5" | Negative | 72.5 |
| South Africa | 60 | 113.4 | Female | No | "2.5" | Positive | 28 |
| Uganda | 50 | 113.6 | Male | No | "2.5" | Negative | 35 |
| Uganda | 40 | 114.4 | Male | No | "2.5" | Negative | 50 |
| Uganda | 70 | 114.6 | Male | No | "2.5" | Negative | 37.52 |
| South Africa | 18 | 114.7 | Female | No | "2.5" | Negative | 70 |
| South Africa | 50 | 115.5 | Male | No | "3.0" | Negative | 52.5 |
| South Africa | 60 | 115.5 | Female | No | "2.5" | Negative | 38.64 |
| South Africa | 50 | 117 | Female | No | "2.5" | Positive | 47.5 |
| South Africa | 60 | 117.93 | Female | No | "2.5" | Negative | 56 |
| South Africa | 30 | 118 | Female | No | "2.5" | Positive | 70 |
| Uganda | 50 | 118.8 | Female | No | "2.5" | Negative | 35 |
| Uganda | 50 | 118.8 | Female | No | "2.5" | Negative | 42 |
| Uganda | 50 | 119.1 | Male | No | "2.5" | Positive | 50 |
| South Africa | 30 | 119.29 | Female | No | "3.0" | Negative | 42 |
| South Africa | 60 | 119.7 | Female | No | "2.5" | Negative | 95 |
| Uganda | 80 | 120.2 | Female | Yes | "2.5" | Negative | 43 |
| Uganda | 50 | 120.5 | Female | No | "2.5" | Negative | 30 |
| Uganda | 20 | 121 | Female | No | "2.5" | Negative | 47.5 |
| South Africa | 60 | 121.4 | Female | No | "2.5" | Negative | 35 |
| South Africa | 40 | 121.4 | Male | No | "2.5" | Positive | 47.5 |
| Uganda | 40 | 122.7 | Male | No | "2.5" | Negative | 37.52 |
| South Africa | 60 | 123 | Male | No | "2.5" | Unknown | 25 |
| Uganda | 60 | 123 | Male | No | "2.5" | Negative | 24.5 |
| Uganda | 70 | 125 | Female | No | "2.5" | Negative | 63 |
| South Africa | 30 | 126 | Female | No | "2.5" | Negative | 62.5 |
| South Africa | 60 | 126.5 | Female | No | "3.0" | Negative | 25 |
| Uganda | 60 | 127 | Female | Yes | "3.0" | Negative | 35 |
| Uganda | 40 | 127.01 | Male | No | "2.5" | Negative | 116 |
| Uganda | 50 | 127.91 | Female | No | "2.5" | Negative | 32 |
| South Africa | 60 | 128.6 | Female | No | "2.5" | Positive | 67.48 |
| Uganda | 60 | 128.6 | Female | No | "2.5" | Negative | 70 |
| Uganda | 30 | 129 | Female | Yes | "2.5" | Negative | 38 |
| South Africa | 50 | 129.7 | Female | No | "2.5" | Negative | 115 |
| South Africa | 40 | 130 | Female | No | "2.5" | Negative | 78.75 |
| South Africa | 50 | 131.4 | Male | No | "2.5" | Negative | 65 |
| Uganda | 50 | 131.4 | Male | No | "2.5" | Negative | 27.51 |
| Uganda | 70 | 132 | Female | No | "2.5" | Unknown | 42.5 |
| Uganda | 40 | 134 | Male | No | "2.5" | Positive | 70 |
| Uganda | 40 | 134.1 | Male | No | "2.5" | Negative | 49.98 |
| South Africa | 50 | 134.6 | Male | No | "2.5" | Negative | 82 |
| South Africa | 60 | 135 | Female | No | "2.5" | Negative | 30 |
| Uganda | 50 | 135 | Female | No | "2.5" | Negative | 121.9 |
| Uganda | 30 | 137.3 | Female | No | "2.5" | Unknown | 38 |
| South Africa | 50 | 137.3 | Male | No | "2.5" | Negative | 80 |
| South Africa | 50 | 139.1 | Male | No | "2.5" | Negative | 40 |
| Uganda | 50 | 139.5 | Male | No | "2.5" | Negative | 55 |
| South Africa | 30 | 143.8 | Male | No | "2.5" | Negative | 56 |
| Uganda | 40 | 144.24 | Male | No | "2.5" | Negative | 32 |
| Uganda | 40 | 145 | Male | No | "2.5" | Negative | 44.63 |
| South Africa | 50 | 145.15 | Male | No | "3.0" | Negative | 70 |
| Uganda | 50 | 145.6 | Male | Yes | "2.5" | Positive | 41.3 |
| South Africa | 50 | 150.3 | Male | No | "2.5" | Negative | 56.56 |
| South Africa | 50 | 151 | Female | No | "2.5" | Negative | 28 |
| Uganda | 40 | 152.3 | Female | No | "2.5" | Negative | 52.5 |
| South Africa | 50 | 155.9 | Male | No | "2.5" | Negative | 57.47 |
| Uganda | 60 | 156.04 | Female | No | "2.5" | Negative | 42 |
| Uganda | 50 | 159.2 | Female | No | "3.0" | Positive | 35 |
| Uganda | 30 | 166.9 | Male | No | "2.5" | Negative | 87.5 |
| Uganda | 50 | 172.7 | Female | No | "2.5" | Positive | 75 |
| South Africa | 50 | NA | Female | No | "2.5" | Negative | 64.17 |
| South Africa | 60 | NA | Female | No | "2.5" | Positive | 30 |
| Uganda | 70 | NA | Male | No | "2.5" | Negative | 40 |
| Uganda | 50 | NA | Female | No | "2.5" | Negative | 12.5 |
| South Africa | 40 | NA | Female | No | "2.5" | Negative | 85 |
| Uganda | 30 | NA | Male | No | "2.5" | Negative | 30 |
| Uganda | 20 | NA | Male | No | "2.5" | Negative | 70 |
| South Africa | 50 | NA | Male | No | "2.5" | Negative | 40.81 |
| Uganda | 60 | NA | Male | Yes | "2.5" | Negative | 35 |
| Uganda | 50 | NA | Male | No | "2.5" | Negative | 40 |
| South Africa | 80 | NA | Male | No | "2.5" | Negative | 52.5 |

**##Example results**

This performance was obtained by fitting the final model in the example dataset and computing the performance. The final parameters shown in Table S2 and the R Code above were used.

| **Technique** | **MAE, (95% CI), mg/wk** | **Unbiased MAPE^a^ (95% CI), %** | **Bias^b^ (95% CI), %** | **% of patients with ideal dose^c^ (95% CI)** | **High under-dosing risk^d^ (95% CI), %** | **Low over-/under-dosing risk^d^ (95% CI), %** | **High over-dosing risk^d^ (95% CI), %** |
| --- | --- | --- | --- | --- | --- | --- | --- |
| Ordinary least squares regression | 12.01 (10.81; 13.22) | 34.97 (31.41; 38.62) | 0.00 (-4.02; 4.18) | 43.50 (38.34; 48.66) | 18.59 (14.57; 22.61) | 72.07 (67.36; 76.78) | 9.34 (6.29; 12.39) |
| Nonlinear least squares regression | 12.02 (10.80; 13.23) | 34.95 (31.39; 38.61) | 0.22 (-3.80; 4.42) | 42.86 (37.72; 48.00) | 18.04 (14.05; 22.03) | 72.89 (68.20; 77.59) | 9.07 (6.05; 12.08) |
| Quantile regression | 11.96 (10.75; 13.17) | 34.81 (31.22; 38.51) | 0.93 (-3.14; 5.18) | 44.23 (39.03; 49.43) | 17.49 (13.57; 21.41) | 73.17 (68.56; 77.78) | 9.34 (6.32; 12.36) |
| Robust regression | 12.00 (10.80; 13.20) | 34.92 (31.35; 38.60) | 1.47 (-2.60; 5.71) | 43.59 (38.48; 48.70) | 17.22 (13.31; 21.12) | 72.89 (68.22; 77.56) | 9.89 (6.81; 12.97) |
| Ridge regression | 12.19 (10.96; 13.43) | 35.57 (31.94; 39.31) | 0.00 (-4.07; 4.25) | 43.13 (37.85; 48.42) | 19.41 (15.43; 23.39) | 71.52 (66.86; 76.18) | 9.07 (6.04; 12.10) |
| LASSO regression | 12.21 (10.99; 13.43) | 35.63 (32.02; 39.34) | 0.00 (-4.06; 4.23) | 42.95 (37.65; 48.24) | 18.86 (14.86; 22.87) | 71.79 (67.08; 76.51) | 9.34 (6.26; 12.43) |
| Elastic net regression | 12.21 (10.99; 13.44) | 35.64 (32.03; 39.35) | 0.00 (-4.06; 4.23) | 43.50 (38.19; 48.81) | 19.14 (15.13; 23.15) | 71.79 (67.11; 76.48) | 9.07 (6.00; 12.13) |
| Principal components regression | 12.01 (10.81; 13.22) | 34.97 (31.41; 38.62) | 0.00 (-4.02; 4.18) | 43.50 (38.34; 48.66) | 18.59 (14.57; 22.61) | 72.07 (67.36; 76.78) | 9.34 (6.29; 12.39) |
| Partial least squares regression | 12.01 (10.80; 13.22) | 34.96 (31.40; 38.60) | 0.00 (-4.02; 4.18) | 43.50 (38.37; 48.63) | 18.86 (14.83; 22.90) | 71.52 (66.79; 76.25) | 9.62 (6.54; 12.69) |
| k-nearest neighbors | 12.96 (11.65; 14.27) | 38.24 (34.36; 42.23) | -0.94 (-5.26; 3.57) | 42.95 (37.71; 48.19) | 20.88 (16.73; 25.03) | 69.51 (64.71; 74.30) | 9.62 (6.51; 12.72) |
| Artificial neural networks | 12.13 (10.93; 13.34) | 35.35 (31.79; 39.01) | 0.00 (-5.10; 5.37) | 41.76 (36.61; 46.91) | 18.32 (13.75; 22.88) | 72.07 (67.25; 76.89) | 9.62 (6.23; 13.00) |
| Support vector regression | 12.14 (10.92; 13.36) | 35.40 (31.81; 39.08) | -0.03 (-4.84; 5.03) | 42.67 (37.47; 47.87) | 17.77 (13.60; 21.93) | 72.89 (68.22; 77.57) | 9.34 (6.06; 12.62) |
| Regression trees | 11.99 (10.78; 13.21) | 34.78 (31.27; 38.38) | 0.00 (-4.01; 4.18) | 44.96 (39.82; 50.11) | 20.15 (16.01; 24.28) | 71.34 (66.66; 76.01) | 8.52 (5.56; 11.47) |
| Model trees | 13.12 (11.79; 14.45) | 40.32 (36.36; 44.40) | -10.48 (-14.35; -6.43) | 40.11 (34.94; 45.28) | 28.66 (23.74; 33.59) | 63.37 (58.15; 68.59) | 7.97 (5.11; 10.82) |
| Bagged trees | 2.94 (2.54; 3.33) | 7.34 (6.46; 8.22) | -0.04 (-1.22; 1.16) | 95.15 (92.91; 97.39) | 0.82 (0.00; 1.93)^e^ | 98.9 (97.65; 100.00)^e^ | 0.27 (0.00; 0.83)^e^ |
| Random forest regression | 11.56 (10.35; 12.78) | 33.27 (29.88; 36.75) | 0.10 (-3.75; 4.11) | 46.43 (41.19; 51.67) | 17.67 (13.84; 21.51) | 74.63 (70.12; 79.15) | 7.69 (4.85; 10.53) |
| Boosted trees | 11.83 (10.64; 13.02) | 34.30 (30.83; 37.86) | -0.07 (-4.01; 4.03) | 44.51 (39.29; 49.72) | 19.23 (15.14; 23.32) | 71.15 (66.43; 75.88) | 9.62 (6.55; 12.68) |
| Bayesian additive regression trees | 11.93 (10.72; 13.15) | 34.47 (30.99; 38.04) | -0.05 (-4.00; 4.07) | 43.86 (38.71; 49.02) | 18.86 (14.75; 22.97) | 73.17 (68.54; 77.80) | 7.97 (5.19; 10.74) |
| Multivariate adaptive regression splines | 12.18 (10.98; 13.38) | 35.50 (32.00; 39.09) | 0.00 (-4.02; 4.18) | 43.13 (38.04; 48.23) | 19.6 (15.46; 23.74) | 70.79 (65.96; 75.61) | 9.62 (6.52; 12.71) |
| Cubist | 8.15 (7.21; 9.09) | 21.80 (19.68; 23.96) | 0.18 (-2.46; 2.89) | 59.07 (53.88; 64.26) | 9.25 (6.26; 12.23) | 87.73 (84.30; 91.15) | 3.02 (1.26; 4.79) |
| eXtreme Gradient Boosting | 11.71 (10.52; 12.90) | 33.95 (30.49; 37.50) | -0.01 (-3.93; 4.07) | 45.05 (39.85; 50.26) | 18.68 (14.64; 22.73) | 71.70 (66.97; 76.43) | 9.62 (6.54; 12.69) |

^a^Unbiased MAPE = (exp(mean(absolute(log(predicted dose/actual dose)))) – 1) $\times$ 100. ^b^Bias = (exp(mean(log(predicted dose/actual dose))) – 1) $\times$ 100 (negative and positive values imply under- and over-estimation respectively). ^c^The ideal dose was defined as the predicted dose within 20% of the actual dose. ^d^Being at risk of under- or over-dosing was defined as respectively having an actual dose at least 40% lower or higher than the predicted dose. ^e^Being proportions, the lower and upper CI were set to 0% and 100% respectively. CI = confidence intervals; LASSO = least absolute shrinkage and selection operator; MAE = mean absolute error; MAPE = mean absolute percentage error; War-PATH = WARfarin anticoagulation in PATients in sub-SaHaran Africa. Based on 3 imputed datasets (the imputation models incorporated all predictor variables and the stable weekly dose).

**Supplementary Tables**

**Table S1. TRIPOD Checklist: Prediction Model Development and Validation**

| **Section/Topic** | **Item** |  | **Checklist Item** | **Section/Pg** |
| --- | --- | --- | --- | --- |
| **Title and abstract** | | | | |
| Title | 1 | D;V | Identify the study as developing and/or validating a multivariable prediction model, the target population, and the outcome to be predicted. | 1 |
| Abstract | 2 | D;V | Provide a summary of objectives, study design, setting, participants, sample size, predictors, outcome, statistical analysis, results, and conclusions. | 1-2^a^ |
| **Introduction** | | | | |
| Background and objectives | 3a | D;V | Explain the medical context (including whether diagnostic or prognostic) and rationale for developing or validating the multivariable prediction model, including references to existing models. | 2 |
|  | 3b | D;V | Specify the objectives, including whether the study describes the development or validation of the model or both. | 3 |
| **Methods** | | | | |
| Source of data | 4a | D;V | Describe the study design or source of data (e.g., randomized trial, cohort, or registry data), separately for the development and validation data sets, if applicable. | 3 |
|  | 4b | D;V | Specify the key study dates, including start of accrual; end of accrual; and, if applicable, end of follow-up. | 3 |
| Participants | 5a | D;V | Specify key elements of the study setting (e.g., primary care, secondary care, general population) including number and location of centres. | 3 |
|  | 5b | D;V | Describe eligibility criteria for participants. | 3 |
|  | 5c | D;V | Give details of treatments received, if relevant. | 3 |
| Outcome | 6a | D;V | Clearly define the outcome that is predicted by the prediction model, including how and when assessed. | 3 |
|  | 6b | D;V | Report any actions to blind assessment of the outcome to be predicted. | NA^a^ |
| Predictors | 7a | D;V | Clearly define all predictors used in developing or validating the multivariable prediction model, including how and when they were measured. | 3 |
|  | 7b | D;V | Report any actions to blind assessment of predictors for the outcome and other predictors. | NA^b^ |
| Sample size | 8 | D;V | Explain how the study size was arrived at. | 3 |
| Missing data | 9 | D;V | Describe how missing data were handled (e.g., complete-case analysis, single imputation, multiple imputation) with details of any imputation method. | 3-4 |
| Statistical analysis methods | 10a | D | Describe how predictors were handled in the analyses. | 4 |
|  | 10b | D | Specify type of model, all model-building procedures (including any predictor selection), and method for internal validation. | 4 |
|  | 10c | V | For validation, describe how the predictions were calculated. | 4 |
|  | 10d | D;V | Specify all measures used to assess model performance and, if relevant, to compare multiple models. | 4-5 |
|  | 10e | V | Describe any model updating (e.g., recalibration) arising from the validation, if done. | NA |
| Risk groups | 11 | D;V | Provide details on how risk groups were created, if done. | 5 |
| Development vs. validation | 12 | V | For validation, identify any differences from the development data in setting, eligibility criteria, outcome, and predictors. | 3, Table 1 |
| **Results** | | | | |
| Participants | 13a | D;V | Describe the flow of participants through the study, including the number of participants with and without the outcome and, if applicable, a summary of the follow-up time. A diagram may be helpful. | Previous study^1^ |
|  | 13b | D;V | Describe the characteristics of the participants (basic demographics, clinical features, available predictors), including the number of participants with missing data for predictors and outcome. | Table 1 |
|  | 13c | V | For validation, show a comparison with the development data of the distribution of important variables (demographics, predictors and outcome). | Table 1 |
| Model development | 14a | D | Specify the number of participants and outcome events in each analysis. | 3 |
|  | 14b | D | If done, report the unadjusted association between each candidate predictor and outcome. | NA |
| Model specification | 15a | D | Present the full prediction model to allow predictions for individuals (i.e., all regression coefficients, and model intercept or baseline survival at a given time point). | NA |
|  | 15b | D | Explain how to the use the prediction model. | NA |
| Model performance | 16 | D;V | Report performance measures (with CIs) for the prediction model. | Tables S3-S4 |
| Model-updating | 17 | V | If done, report the results from any model updating (i.e., model specification, model performance). | NA |
| **Discussion** | | | | |
| Limitations | 18 | D;V | Discuss any limitations of the study (such as nonrepresentative sample, few events per predictor, missing data). | 8-9 |
| Interpretation | 19a | V | For validation, discuss the results with reference to performance in the development data, and any other validation data. | 7-8 |
|  | 19b | D;V | Give an overall interpretation of the results, considering objectives, limitations, results from similar studies, and other relevant evidence. | 7-9 |
| Implications | 20 | D;V | Discuss the potential clinical use of the model and implications for future research. | 8-9 |
| **Other information** | | | | |
| Supplementary information | 21 | D;V | Provide information about the availability of supplementary resources, such as study protocol, Web calculator, and data sets. | 11 |
| Funding | 22 | D;V | Give the source of funding and the role of the funders for the present study. | 1 |

^a^Some details omitted due to word limitations.  ^b^Not applicable. The predictors (clinical and demographic variables such as age, sex, target INR range, HIV status, weight, country of recruitment, concomitant medications etc.) are usually captured before the first warfarin dose is given to the patient and it will be days before the stable dose (the outcome) is attained. Even when measured after, all the predictors and outcome variable, as defined in this study, are objective measures and so would not benefit from blinding procedures.

Items relevant only to the development of a prediction model are denoted by D, items relating solely to a validation of a prediction model are denoted by V, and items relating to both are denoted D;V. We recommend using the TRIPOD Checklist in conjunction with the TRIPOD Explanation and Elaboration document.^2^

**Table S2. Machine learning techniques implemented in the current study^a^**

| **Technique** | **R package** | **Scaling** | **Tuned parameters (value used)** |
| --- | --- | --- | --- |
| Ordinary least squares regression | stats^3^ | No | None^b^ |
| Nonlinear least squares regression | stats^3^ | No | None^b^ |
| Quantile regression | quantreg^4^ | No | None^b^ |
| Robust regression | MASS^5^ | No | None^b^ |
| Ridge regression (alpha = 0) | glmnet^6^ | Yes | Lambda (0.2945) |
| LASSO regression (alpha = 1) | glmnet^6^ | Yes | Lambda (0.0309) |
| Elastic net regression (alpha = 0.5) | glmnet^6^ | Yes | Lambda (0.0562) |
| Principal components regression | pls^7^ | Yes | Number of principal components (8)^c^ |
| Partial least squares regression | pls^7^ | Yes | Number of principal components (2) |
| k-nearest neighbors | FNN^8^ | Yes | Number of nearest neigbours (566) |
| Artificial neural networks | neuralnet^9^ | Yes | Hidden layers and nodes (1 hidden layer, 1 hidden node) |
| Support vector regression | e1071^10^ | Yes | Kernel (polynomial), degree (1), gamma (0.1), cost (1), epsilon (0.1) |
| Regression trees | tree^11^ | No | Tree size/number of terminal nodes (4) |
| Model trees | RWeka^12,13^ | No | None^d^ |
| Bagged trees | randomForest^14^ | No | Number of trees (500), minimum size of terminal nodes (8), mtry (7) |
| Random forest regression | randomForest^14^ | No | Number of trees (500), minimum size of terminal nodes (8), mtry (1) |
| Boosted trees | gbm^15^ | No | Number of trees (500), n.minobsinnode (2), shrinkage (0.01) |
| Bayesian additive regression trees | bartmachine^16^ | No | Number of trees (1) |
| Multivariate adaptive regression splines | earth^17^ | No | Maximum number of terms (including intercept) in the pruned mode (4) |
| Cubist | Cubist^18^ | No | Committees (100) |
| eXtreme Gradient Boosting | xgboost^19^ | No | Booster (gbtree), eta (0.1), gamma (0), max_depth (1), nrounds (100) |

^a^All analysis conducted in R version 4.0.2^20^. ^b^Coefficients were shrunk to avoid overfitting the development dataset. ^c^Equivalent to ordinary least squares regression since all principal components were used.  ^d^Tree pruning is incorporated in the model-fitting process. LASSO = least absolute shrinkage and selection operator.

**Table S3.** **Performance of the various algorithms** **in the War-PATH development cohort (*N* = 364)**

| **Technique** | **MAE, (95% CI), mg/wk** | **Unbiased MAPE^a^ (95% CI), %** | **Bias^b^ (95% CI), %** | **% of patients with ideal dose^c^ (95% CI)** | **High under-dosing risk^d^ (95% CI), %** | **Low over-/under-dosing risk^d^ (95% CI), %** | **High over-dosing risk^d^ (95% CI), %** |
| --- | --- | --- | --- | --- | --- | --- | --- |
| Fixed (35 mg/wk) | 12.30 (10.82; 13.77) | 36.52 (32.46; 40.71) | -5.79 (-9.74; -1.68) | 43.96 (38.78; 49.13) | 26.65 (21.90; 31.40) | 64.56 (59.35; 69.77) | 8.79 (5.88; 11.70) |
| War-PATH | 11.57 (10.35; 12.79) | 33.94 (30.62; 37.36) | 0.23 (-3.54; 4.13) | 41.48 (36.42; 46.55) | 19.87 (15.72; 24.02) | 72.16 (67.33; 76.99) | 7.97 (5.14; 10.80) |
| Ordinary least squares regression | 11.50 (10.30; 12.70) | 33.66 (30.37; 37.04) | 0.00 (-3.71; 3.85) | 41.12 (36.01; 46.22) | 17.95 (13.97; 21.93) | 73.81 (69.14; 78.48) | 8.24 (5.41; 11.08) |
| Nonlinear least squares regression | 11.44 (10.23; 12.65) | 33.47 (30.17; 36.85) | 0.26 (-3.46; 4.12) | 41.94 (36.88; 47.00) | 18.22 (14.13; 22.31) | 74.18 (69.44; 78.91) | 7.60 (4.87; 10.33) |
| Quantile regression | 11.38 (10.14; 12.62) | 33.33 (29.93; 36.82) | 0.67 (-3.10; 4.59) | 44.32 (39.09; 49.56) | 19.14 (15.09; 23.19) | 71.25 (66.43; 76.06) | 9.62 (6.56; 12.67) |
| Robust regression | 11.44 (10.23; 12.65) | 33.48 (30.16; 36.89) | 0.72 (-3.02; 4.61) | 43.86 (38.73; 49.00) | 17.4 (13.42; 21.37) | 74.08 (69.36; 78.81) | 8.52 (5.58; 11.45) |
| Ridge regression | 11.48 (10.21; 12.75) | 33.65 (30.20; 37.18) | 0.00 (-3.79; 3.94) | 43.96 (38.68; 49.23) | 20.15 (16.01; 24.28) | 69.69 (64.88; 74.5) | 10.16 (7.08; 13.25) |
| LASSO regression | 11.51 (10.25; 12.77) | 33.73 (30.33; 37.23) | 0.00 (-3.79; 3.94) | 42.86 (37.64; 48.08) | 19.14 (15.10; 23.17) | 70.70 (65.95; 75.44) | 10.16 (7.08; 13.25) |
| Elastic net regression | 11.51 (10.25; 12.77) | 33.74 (30.32; 37.25) | 0.00 (-3.79; 3.94) | 43.13 (37.91; 48.36) | 19.14 (15.10; 23.17) | 70.70 (65.95; 75.44) | 10.16 (7.08; 13.25) |
| Principal components regression | 11.50 (10.30; 12.70) | 33.66 (30.37; 37.04) | 0.00 (-3.71; 3.85) | 41.12 (36.01; 46.22) | 17.95 (13.97; 21.93) | 73.81 (69.14; 78.48) | 8.24 (5.41; 11.08) |
| Partial least squares regression | 11.48 (10.27; 12.69) | 33.64 (30.32; 37.05) | 0.00 (-3.72; 3.86) | 42.58 (37.36; 47.81) | 18.41 (14.37; 22.45) | 73.26 (68.52; 78.00) | 8.33 (5.43; 11.23) |
| k-nearest neighbors | 11.91 (10.55; 13.27) | 35.10 (31.39; 38.92) | -2.13 (-6.06; 1.96) | 44.23 (38.94; 49.52) | 22.07 (17.70; 26.44) | 67.77 (62.78; 72.75) | 10.16 (7.10; 13.23) |
| Artificial neural networks | 11.52 (10.31; 12.72) | 33.72 (30.49; 37.04) | 0.00 (-4.69; 4.92) | 42.49 (37.36; 47.62) | 17.40 (11.73; 23.07) | 75.18 (69.87; 80.49) | 7.42 (4.35; 10.48) |
| Support vector regression | 11.46 (10.25; 12.67) | 33.59 (30.21; 37.05) | 1.74 (-2.49; 6.14) | 44.05 (38.82; 49.27) | 18.13 (13.77; 22.49) | 71.98 (67.17; 76.78) | 9.89 (6.60; 13.18) |
| Regression trees | 11.66 (10.47; 12.85) | 34.42 (31.21; 37.73) | 0.00 (-3.82; 3.97) | 41.67 (36.38; 46.95) | 18.77 (14.78; 22.76) | 71.61 (66.96; 76.27) | 9.62 (6.48; 12.75) |
| Model trees | 11.14 (10.25; 12.03) | 32.46 (29.52; 35.46) | 17.93 (14.25; 21.73) | 45.79 (40.71; 50.87) | 5.31 (3.00; 7.63) | 80.59 (76.40; 84.77) | 14.10 (10.57; 17.64) |
| Bagged trees | 2.26 (1.95; 2.56) | 5.64 (5.06; 6.20) | -0.09 (-0.87; 0.70) | 97.34 (95.66; 99.03) | 0.09 (0.00; 0.56)^e^ | 99.91 (99.44; 100.00)^e^ | 0.00 (0.00; 0.00)^e^ |
| Random forest regression | 10.58 (9.39; 11.76) | 30.39 (27.39; 33.47) | -0.02 (-3.46; 3.53) | 46.43 (41.16; 51.70) | 17.31 (13.46; 21.15) | 75.00 (70.56; 79.44) | 7.69 (4.99; 10.39) |
| Boosted trees | 11.30 (10.07; 12.52) | 32.90 (29.68; 36.19) | 0.04 (-3.61; 3.83) | 44.96 (39.69; 50.24) | 18.22 (14.24; 22.21) | 72.99 (68.33; 77.64) | 8.79 (5.89; 11.69) |
| Bayesian additive regression trees | 11.05 (9.93; 12.16) | 32.24 (29.37; 35.17) | -0.08 (-3.57; 3.54) | 46.61 (41.56; 51.66) | 15.48 (11.71; 19.24) | 79.03 (74.76; 83.30) | 5.49 (3.11; 7.88) |
| Multivariate adaptive regression splines | 11.63 (10.42; 12.83) | 34.13 (30.83; 37.52) | 0.00 (-3.76; 3.90) | 42.58 (37.43; 47.73) | 18.04 (14.06; 22.02) | 73.72 (69.05; 78.39) | 8.24 (5.37; 11.11) |
| Cubist | 6.98 (6.20; 7.76) | 18.76 (17.06; 20.48) | 0.44 (-1.76; 2.71) | 65.02 (60.14; 69.90) | 5.49 (3.08; 7.91) | 92.58 (89.72; 95.44) | 1.92 (0.48; 3.37) |
| eXtreme Gradient Boosting | 11.15 (9.95; 12.35) | 32.38 (29.25; 35.58) | -0.01 (-3.58; 3.71) | 44.78 (39.46; 50.10) | 17.12 (13.24; 21.01) | 75.18 (70.59; 79.78) | 7.69 (4.95; 10.43) |

^a^Unbiased MAPE = (exp(mean(absolute(log(predicted dose/actual dose)))) – 1) $\times$ 100. ^b^Bias = (exp(mean(log(predicted dose/actual dose))) – 1) $\times$ 100 (negative and positive values imply under- and over-estimation respectively). ^c^The ideal dose was defined as the predicted dose within 20% of the actual dose. ^d^Being at risk of under- or over-dosing was defined as respectively having an actual dose at least 40% lower or higher than the predicted dose. ^e^Being proportions, the lower and upper CI were set to 0% and 100% respectively. CI = confidence intervals; LASSO = least absolute shrinkage and selection operator; MAE = mean absolute error; MAPE = mean absolute percentage error; War-PATH = WARfarin anticoagulation in PATients in sub-SaHaran Africa. Based on 3 imputed datasets (the imputation models incorporated all predictor variables and the stable weekly dose).

**Table S4. Performance of the various algorithms in the War-****PATH external validation cohort (*N* = 270)**

| **Technique** | **MAE, (95% CI), mg/wk** | **Unbiased MAPE^a^ (95% CI), %** | **Bias^b^ (95% CI), %** | **% of patients with ideal dose^c^ (95% CI)** | **High under-dosing risk^d^ (95% CI), %** | **Low over-/under-dosing risk^d^ (95% CI), %** | **High over-dosing risk^d^ (95% CI), %** |
| --- | --- | --- | --- | --- | --- | --- | --- |
| Fixed (35 mg/wk) | 11.91 (10.04; 13.78) | 35.66 (30.92; 40.57) | -3.34 (-8.38; 1.97) | 50.37 (44.52; 56.22) | 20.00 (15.12; 24.88) | 68.89 (63.44; 74.34) | 11.11 (7.30; 14.92) |
| War-PATH | 12.57 (10.99; 14.15) | 38.21 (33.80; 42.78) | 3.84 (-1.40; 9.36) | 43.33 (37.32; 49.35) | 16.30 (11.87; 20.72) | 69.26 (63.94; 74.58) | 14.44 (10.34; 18.55) |
| Ordinary least squares regression | 13.01 (11.45; 14.58) | 39.68 (35.16; 44.34) | 4.66 (-0.70; 10.31) | 42.59 (36.74; 48.45) | 16.30 (11.89; 20.70) | 68.89 (63.62; 74.16) | 14.81 (10.73; 18.90) |
| Nonlinear least squares regression | 12.78 (11.20; 14.37) | 39.01 (34.49; 43.69) | 4.53 (-0.81; 10.15) | 43.33 (37.48; 49.19) | 15.56 (11.20; 19.91) | 69.63 (64.38; 74.88) | 14.81 (10.68; 18.95) |
| Quantile regression | 12.39 (10.79; 13.98) | 37.42 (32.96; 42.03) | 5.31 (0.01; 10.88) | 48.52 (42.57; 54.47) | 14.44 (10.17; 18.72) | 71.48 (66.22; 76.74) | 14.07 (9.89; 18.26) |
| Robust regression | 12.81 (11.25; 14.37) | 38.94 (34.46; 43.56) | 5.44 (0.09; 11.07) | 44.44 (38.54; 50.34) | 14.81 (10.52; 19.11) | 70.00 (64.77; 75.23) | 15.19 (10.96; 19.41) |
| Ridge regression | 12.19 (10.54; 13.84) | 36.80 (32.27; 41.48) | 4.22 (-1.04; 9.77) | 48.15 (42.26; 54.04) | 15.19 (10.80; 19.57) | 71.11 (65.84; 76.39) | 13.70 (9.55; 17.86) |
| LASSO regression | 12.29 (10.65; 13.93) | 37.06 (32.54; 41.74) | 4.10 (-1.18; 9.67) | 49.26 (43.35; 55.17) | 15.19 (10.80; 19.58) | 70.74 (65.42; 76.06) | 14.07 (9.98; 18.16) |
| Elastic net regression | 12.27 (10.63; 13.91) | 37.01 (32.49; 41.69) | 4.15 (-1.14; 9.72) | 50.00 (44.11; 55.89) | 14.81 (10.45; 19.18) | 71.11 (65.81; 76.42) | 14.07 (9.98; 18.16) |
| Principal components regression | 13.01 (11.45; 14.58) | 39.68 (35.16; 44.34) | 4.66 (-0.70; 10.31) | 42.59 (36.74; 48.45) | 16.30 (11.89; 20.70) | 68.89 (63.62; 74.16) | 14.81 (10.73; 18.90) |
| Partial least squares regression | 13.11 (11.54; 14.68) | 40.06 (35.49; 44.79) | 5.19 (-0.22; 10.88) | 41.85 (35.94; 47.76) | 15.56 (11.19; 19.92) | 69.26 (63.99; 74.53) | 15.19 (10.99; 19.38) |
| k-nearest neighbors | 12.12 (10.76; 13.48) | 36.47 (32.71; 40.34) | 1.46 (-2.62; 5.71) | 49.63 (44.35; 54.91) | 16.67 (12.29; 21.04) | 71.11 (66.13; 76.09) | 12.22 (9.16; 15.29) |
| Artificial neural networks | 13.01 (11.81; 14.21) | 40.18 (36.79; 43.65) | 3.11 (-1.72; 8.18) | 40.74 (35.62; 45.86) | 18.52 (12.88; 24.16) | 67.78 (62.52; 73.03) | 13.70 (10.58; 16.83) |
| Support vector regression | 12.92 (11.71; 14.12) | 39.26 (35.74; 42.86) | 5.80 (1.41; 10.38) | 43.33 (38.16; 48.51) | 14.07 (9.64; 18.51) | 70.00 (65.18; 74.82) | 15.93 (12.64; 19.21) |
| Regression trees | 12.52 (10.91; 14.14) | 37.71 (33.27; 42.29) | 4.49 (-0.84; 10.11) | 44.44 (38.53; 50.36) | 16.67 (12.25; 21.08) | 71.11 (65.82; 76.40) | 12.22 (8.41; 16.03) |
| Model trees | 17.59 (15.75; 19.43) | 51.18 (45.31; 57.30) | 24.65 (17.43; 32.31) | 31.48 (25.87; 37.09) | 9.63 (6.06; 13.2) | 64.81 (59.23; 70.40) | 25.56 (20.36; 30.75) |
| Bagged trees | 14.67 (13.05; 16.29) | 44.41 (39.63; 49.36) | 7.18 (1.29; 13.42) | 35.19 (29.43; 40.94) | 17.41 (12.73; 22.09) | 67.41 (61.72; 73.10) | 15.19 (10.89; 19.48) |
| Random forest regression | 12.07 (10.39; 13.76) | 36.30 (31.76; 41.00) | 4.33 (-0.94; 9.88) | 48.52 (42.56; 54.48) | 15.19 (10.81; 19.56) | 71.11 (65.78; 76.45) | 13.70 (9.56; 17.85) |
| Boosted trees | 12.17 (10.55; 13.79) | 36.72 (32.29; 41.31) | 4.30 (-0.93; 9.80) | 47.04 (41.03; 53.04) | 14.81 (10.49; 19.14) | 71.48 (66.24; 76.73) | 13.70 (9.71; 17.70) |
| Bayesian additive regression trees | 12.72 (11.16; 14.27) | 38.36 (34.10; 42.76) | 4.13 (-1.08; 9.64) | 47.41 (41.65; 53.17) | 16.30 (11.76; 20.83) | 71.48 (65.96; 77.00) | 12.22 (8.30; 16.15) |
| Multivariate adaptive regression splines | 12.99 (11.41; 14.58) | 39.49 (34.93; 44.21) | 4.45 (-0.95; 10.15) | 44.81 (38.98; 50.65) | 16.67 (12.21; 21.12) | 67.41 (61.98; 72.84) | 15.93 (11.65; 20.2) |
| Cubist | 13.41 (11.77; 15.05) | 40.88 (36.14; 45.78) | 5.67 (0.10; 11.55) | 42.22 (36.25; 48.19) | 17.04 (12.45; 21.63) | 67.41 (61.82; 73.00) | 15.56 (11.32; 19.79) |
| eXtreme Gradient Boosting | 12.35 (10.75; 13.94) | 37.39 (32.98; 41.95) | 4.31 (-0.94; 9.82) | 45.56 (39.59; 51.52) | 15.93 (11.52; 20.33) | 70.37 (65.04; 75.70) | 13.70 (9.66; 17.75) |

^a^Unbiased MAPE = (exp(mean(absolute(log(predicted dose/actual dose)))) – 1) $\times$ 100. ^b^Bias = (exp(mean(log(predicted dose/actual dose))) – 1) $\times$ 100 (negative and positive values imply under- and over-estimation respectively). ^c^The ideal dose was defined as the predicted dose within 20% of the actual dose. ^d^Being at risk of under- or over-dosing was defined as respectively having an actual dose at least 40% lower or higher than the predicted dose. CI = confidence intervals; LASSO = least absolute shrinkage and selection operator; MAE = mean absolute error; MAPE = mean absolute percentage error; War-PATH = WARfarin anticoagulation in PATients in sub-SaHaran Africa. Based on 3 imputed datasets (the imputation models incorporated all predictor variables and the stable weekly dose).

**Supplementary References**

1. Asiimwe IG, Waitt C, Sekaggya-Wiltshire C, et al. Developing and Validating a Clinical Warfarin Dose-Initiation Model for Black-African Patients in South Africa and Uganda. *Clin Pharmacol Ther.* 2020.

2. Moons KG, Altman DG, Reitsma JB, et al. Transparent Reporting of a multivariable prediction model for Individual Prognosis or Diagnosis (TRIPOD): explanation and elaboration. *Ann Intern Med.* 2015;162(1):W1-73.

3. *R: A language and environment for statistical computing.* [computer program]. Vienna: R Foundation for Statistical Computing; 2019.

4. *quantreg: Quantile Regression.* [computer program]. 2019.

5. Venables WN, Ripley BD. *Modern Applied Statistics with S.* Fourth ed. New York: Springer; 2002.

6. Friedman J, Hastie T, Tibshirani R. Regularization Paths for Generalized Linear Models via Coordinate Descent. . *Journal of Statistical Software.* 2010;33(1):1-22.

7. Mevik B-H, Wehrens R, Liland KH. Partial Least Squares and Principal Component Regression. R package version 2.7-2. <https://CRAN.R-project.org/package=pls>. Published 2019. Accessed.

8. Beygelzimer A, Kakadet S, Langford J, Arya S, Mount D, Shengqiao Li S. FNN: Fast Nearest Neighbor Search Algorithms and Applications. R package version 1.1.3. <https://CRAN.R-project.org/package=FNN>. Published 2019. Accessed.

9. Fritsch S, Guenther F, Wright MN. neuralnet: Training of Neural Networks. R package version 1.44.2. <https://CRAN.R-project.org/package=neuralnet>. Published 2019. Accessed.

10. Meyer D, Dimitriadou E, Hornik K, Weingessel A, Leisch F. e1071: Misc Functions of the Department of Statistics, Probability Theory Group (Formerly: E1071), TU Wien. R package version 1.7-3. <https://CRAN.R-project.org/package=e1071>. Published 2019. Accessed.

11. Ripley B. tree: Classification and Regression Trees. R package version 1.0-40. <https://CRAN.R-project.org/package=tree>. Published 2019. Accessed.

12. Hornik K, Buchta C, Zeileis A. Open-Source Machine Learning: R Meets Weka. *Computational Statistics.* 2009;24(2):225-232.

13. Witten IH, Frank E. *Data Mining: Practical Machine Learning Tools and Techniques.* 2nd ed. San Francisco: Morgan Kaufmann; 2005.

14. Liaw A, Wiener M. Classification and Regression by randomForest. *R News.* 2002;2(3):18-22.

15. Greenwell B, Boehmke B, Cunningham J, GBM Developers. gbm: Generalized Boosted Regression Models. R package version 2.1.8. <https://CRAN.R-project.org/package=gbm>. Published 2020. Accessed.

16. Kapelner A, Bleich J. bartMachine: Machine Learning with Bayesian Additive Regression Trees. *Journal of Statistical Software.* 2016;20(4):1-40.

17. Milborrow S. earth: Multivariate Adaptive Regression Splines. R package version 5.2.0. <https://CRAN.R-project.org/package=earth>. Published 2020. Accessed.

18. Kuhn M, Quinlan R. Cubist: Rule- And Instance-Based Regression Modeling. R package version 0.2.3. <https://CRAN.R-project.org/package=Cubist>. Published 2020. Accessed.

19. *xgboost: Extreme Gradient Boosting. R package version 1.3.2.1.* [computer program]. 2021.

20. *R: A language and environment for statistical computing. R Foundation for Statistical Computing.* [computer program]. Vienna, Austria2020.
